# Action Graphs for Performing Goal Recognition Design on Human-Inhabited Environments †

**DOI:** 10.3390/s19122741

**Published:** 2019-06-18

**Authors:** Helen Harman, Pieter Simoens

**Affiliations:** Department of Information Technology—IDLab, Ghent University—imec, Technologiepark 126, B-9052 Ghent, Belgium; pieter.simoens@ugent.be

**Keywords:** goal recognition design, symbolic AI, intention recognition, human aware, graph algorithms, modelling actions, redesigning environments, context-awareness, increasing distinctiveness

## Abstract

Goal recognition is an important component of many context-aware and smart environment services; however, a person’s goal often cannot be determined until their plan nears completion. Therefore, by modifying the state of the environment, our work aims to reduce the number of observations required to recognise a human’s goal. These modifications result in either: Actions in the available plans being replaced with more distinctive actions; or removing the possibility of performing some actions, so humans are forced to take an alternative (more distinctive) plan. In our solution, a symbolic representation of actions and the world state is transformed into an Action Graph, which is then traversed to discover the non-distinctive plan prefixes. These prefixes are processed to determine which actions should be replaced or removed. For action replacement, we developed an exhaustive approach and an approach that shrinks the plans then reduces the non-distinctive plan prefixes, namely Shrink–Reduce. Exhaustive is guaranteed to find the minimal distinctiveness but is more computationally expensive than Shrink–Reduce. These approaches are compared using a test domain with varying amounts of goals, variables and values, and a realistic kitchen domain. Our action removal method is shown to increase the distinctiveness of various grid-based navigation problems, with a width/height ranging from 4 to 16 and between 2 and 14 randomly selected goals, by an average of 3.27 actions in an average time of 4.69 s, whereas a state-of-the-art approach often breaches a 10 min time limit.

## 1. Introduction

Through the deployment of numerous IoT sensors, smart environments can attempt to recognise the goal of a human from the actions they perform, and thus become more context-aware. Despite recent advances in Goal Recognition (GR) techniques [1,2], a person’s goal often cannot be determined until their plan nears completion. This is because the plans to reach different goals can initially be identical. Nonetheless, recognising a human’s goal promptly is important in many situations. In environments where security is essential, such as airports [3], improved goal distinctiveness can allow security personnel to intercept a person sooner. In a kitchen environment [4,5], by minimising the number of observations required to recognise a human’s goal, a robot can provide earlier assistance [6].

By redesigning an environment, our work aims to improve the distinctiveness of goals. We have identified two ways Goal Recognition Design (GRD) can affect human-inhabited environments. First, actions in the available plans can be replaced, e.g., by changing an item’s location, the act of taking it from its first location is replaced with taking it from a different location. Second, the possibility of performing an action can be removed, e.g., a barrier or ornament can be placed to prevent a human from navigating between two positions. The resulting environment design potentially improves the accuracy of GR approaches, such as [7,8,9], and requires fewer distinct actions to be detected. In some cases, this reduction could lead to fewer, cheaper or less privacy invasive sensors being deployed in context-aware smart environments.

**Figure 1:**
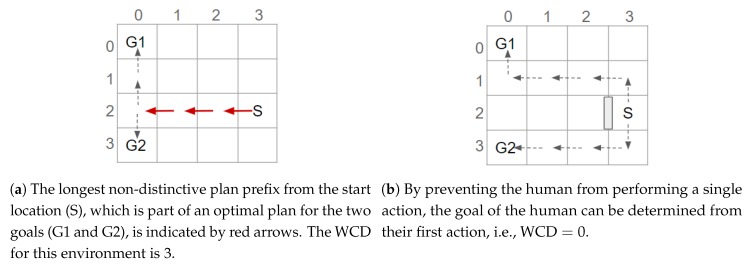
In this grid-based navigation example, a human can move horizontally and vertically. There are multiple optimal plans to each of the goals, but for readability only a single plan to each goal is shown (indicated with arrows). On the left figure, the plans with the longest non-distinctive prefix are displayed. Reproduced from prior work [11].

To clarify the principle of GRD, an example is provided. Figure 1 shows two potential goals. These goals could indicate the locations of the gates in an airport [10]. To recognise which goal a human is aiming to reach, their actions are observed; however, depending on which route is taken, initially the human’s goal cannot be determined. At worst the plans to reach these goals have a non-distinctive prefix containing 3 actions, i.e., the Worst Case Distinctiveness (WCD) is 3. By placing an obstacle to prevent a person from moving between positions (3,2) and (2,2), the WCD of the environment is reduced to 0. In other words, after the environment is redesigned only 1 (discrete) action needs to be observed before the human’s goal is discernible. The term goal recognition design and the WCD metric were introduced by Keren et al. [10].

GRD is a more complex problem than task planning. In task planning the aim is (normally) to find an optimal plan to reach a goal, whereas in GRD there are multiple goals defined and all optimal plans containing non-distinctive prefixes must be found before the environment can be redesigned. Current approaches to GRD [12,13,14,15] usually focus on removing the ability to perform actions or placing order constraints on the actions. To our knowledge, this paper is the first to propose state changes that cause actions in the plans to be replaced, which could result in the length of a plan as well as its non-distinctive prefix changing. As the lengths of the plans can change, we propose a new metric, complementary to WCD, to measure the distinctiveness of an environment.

**Figure 2:**
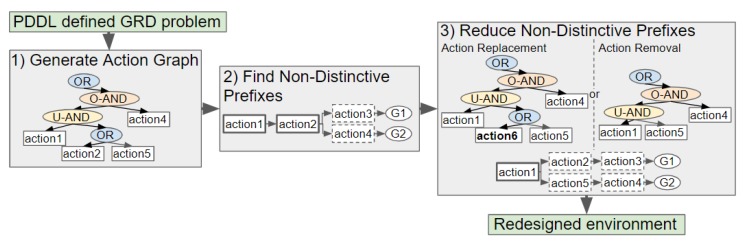
Conceptual overview of our novel approach to goal recognition design (GRD). An Action Graph is created from a GRD problem defined in PDDL. This Action Graph is traversed to identify non-distinctive prefixes. In the third step, modifications are performed to reduce the non-distinctive prefixes, leading to a redesigned environment with an increased goal distinctiveness.

Our novel approach to GRD transforms a problem defined in Planning Domain Definition Language (PDDL), a popular domain-independent language to model the behaviour of deterministic agents, into an Action Graph. Non-distinctive plan prefixes are extracted from the graph, and processed to determine which actions should be removed or replaced to increase the goals’ distinctiveness. Two methods for selecting which actions to replace, namely exhaustive and a less computationally expensive method Shrink–Reduce, are introduced and compared to one another. An overview of our approach is provided in Figure 2.

In our previous work [11], we described a new method for generating an Action Graph for navigation problems. In the current paper, we introduce a new distinctiveness metric, expand the graph creation method to domains with fewer constraints on the order of actions, provide a new algorithm to find the non-distinctive prefixes, and develop a method to change the state of the environment so that actions are replaced. Our approach is not applicable to domains in which cycles exist within the goals’ plans, as Action Graphs are acyclic and no action is repeated within its structure. Moreover, the world is assumed to be fully observable; however, after redesigning the environment fewer actions tend to require observing. The algorithms are evaluated on a grid-based navigation domain, and on a kitchen domain developed by Ramírez and Geffner [7] from the work of Wu et al. [16]. While a domain from [7] is used, a comparison to their approach is not provided as their approach performs GR and not GRD.

Section 2 provides an overview of related work. Section 3 states the formal definition of GRD problems and briefly mentions the GR methods that inspired our approach. The metrics to measure the distinctiveness of an environment are presented in Section 4. Section 5 describes the structure and construction of an Action Graph. The algorithm to find all the non-distinctive plan prefixes in an Action Graph is introduced in Section 6. Section 7 describes how the actions to replace are selected. How actions are removed to increase the distinctiveness is described in Section 8. Finally, we present experimental results in Section 9, by measuring the computational efficiency of our Shrink–Reduce action replacement heuristic and comparing our action removal method to a state-of-the-art approach [10].

## 2. Related Work

The term goal recognition design was coined by Keren et al. [10]. In their approach the WCD of a problem is calculated by transforming a GRD problem into multiple planning problems containing pairs of goals. An optimal plan, with the longest possible non-distinctive prefix, to each pair of goals is searched for. The longest non-distinctive plan prefix, across all joint plans, is the WCD. To reduce WCD an increasing number of actions are removed until either the WCD is 0, or the search space has been exhausted, in which case the best environment design discovered so far is returned. Their pruned-reduce method improves the computational efficiency by reducing the number of action combinations whose removal requires testing. We compare our solution to their pruned-reduce algorithm, and show that for navigation domains we have greatly reduced the time required to solve goal recognition design problems. Further to removing actions, their approach has been extended to action conditioning, in which a partial ordering is enforced [15]. We do not investigate enforcing action ordering, as our focus is on human-inhabited environments, and it is difficult to force action order constraints on humans; but further to their work, we explore domains in which modifications to the state cause actions within a plan to be replaced and that result in the plan’s length changing. Their approach has also been extended for non-optimal agents [17], and to determine where to place sensors within the environment [18]. In this paper we assume the agent/human is optimal and do not investigate sensor placement.

Wayllace et al. [12,14] investigate goal recognition design involving stochastic action outcomes with Markov Decision Processes (MDPs). To calculate WCD, a MDP is created for each goal, the states that are common to pairs of goals are discovered and the Bellman equation is used to calculate the cost of reaching a state. To reduce the WCD their algorithm removes a set of actions, checks that the optimal cost to reach a goal has not been affected, and calculates the WCD to find out if it has been reduced. Their approach creates a MDP multiple times for each of the goals, which results in large computational costs. In our approach a single model (Action Graph) is created, which contains the actions to reach all goals, moreover it is only created once.

In [19], a new metric is introduced, namely expected-case distinctiveness (ECD), which is applicable to stochastic domains and solves a shortcoming of WCD. WCD only incorporates knowledge of the least distinctive pair of goals, rather than all goals’ distinctiveness. To solve this, ECD is calculated recursively, starting from the actions applicable to the initial state and ending with the actions furthest from that state (i.e., that result in a goal state). The resulting ECD is the sum of all weighted plan lengths, with the weights based on prior probabilities of an agent choosing a certain goal. We also introduce a new metric which addresses the mentioned shortcoming of WCD, but for a deterministic setting. Moreover, our metric also accounts for the length of both the plan and non-distinctive prefix being altered during the environment design process.

Son et al. [13] propose an approach based on Answer Set Programming (ASP), as an alternative to PDDL, to reduce the computational cost. Their results only show a maximum of two actions being removed, which greatly limits how much WCD can be reduced. Our approach takes PDDL as input, as we build on the work from [20], which performed well (i.e., computational time and accuracy) on goal recognition problems.

Planning libraries were provided as input to the goal recognition design method by Mirsky et al. [21]. Further, they introduce plan recognition design, in which the aim is to make the plans, rather than goals, distinctive. They present a brute-force method, which gradually removes a higher number of rules from the planning library, and a constraint-based search method. Their constraint-based search attempts to remove different combinations of rules within the non-distinctive plans starting from the rules contained within the least distinctive plans. Like Mirsky et al. [21] and Keren et al. [15], we developed an exhaustive strategy and less computationally expensive method but, rather than preventing actions, removing rules or constraining the order actions must be performed in, our approach changes the state of the environment in such a way that the actions within plans are replaced by others (with the same effects), which could affect the length of the plans as well as the non-distinctive prefixes.

## 3. Background

To clarify the similarities and differences between symbolic task planning, goal recognition and goal recognition design problems, their formal definitions are provided below. The formal notation will be stated during the description of our GRD method. As our work is closely related to GR, this section also introduces the GR approaches that inspired our solution to GRD.

### 3.1. Formal Definition

Task planners (e.g., Fast Downward [22]) search for a sequence of actions (i.e., task plan) that changes the current world state into a desired goal state. Formally, a planning problem *P* can be defined as P=(F,I,A,G), where *F* is a set of fluents, I⊆F is the initial state, G⊆F is a goal state, and *A* is a set of actions along with their preconditions $a_{pre}\subseteq F$ and effects $\left(a_{add}\subseteq F,a_{del}\subseteq F\right)$ [7,10,23,24]. Actions add and delete fluents from states, and action *a* is applicable to the state *s* if $a_{pre}\subseteq s$. In this paper, effects are denoted $a_{eff}$, fluents are interpreted as variables with values, and thus actions change the values of variables, e.g., change the value of a variable that represents a human’s location, or change variables that indicate which items have been taken from cupboards from false to true.

GR is often viewed as the inverse of planning, i.e., T=(F,I,A,O,G) where G is the set of all possible goals and *O* is a sequence of observations [7,25]. Similarly, a GRD problem can be defined as D=(F,I,A,G) [10]. The aim of our GRD method is to find the world model (*I*) that maximises the distinctiveness of the goals G, by reducing the length of the non-distinctive plan prefixes. This results in fewer observations (|O|) being required to determine which of the goals (G) a human is intending to reach.

### 3.2. Goal Recognition

Activity recognition [16], plan recognition [26] and goal recognition [7,8] can all be referred to as intention recognition; nevertheless, their aims slightly differ. Activity recognition labels sensor data with which activity (or action) a human is currently performing, e.g., switching on a kettle. While several previous works have used the terms plan and goal recognition interchangeably, we consider them to be distinct. A plan recogniser attempts to discover the sequence of actions (or possibly hierarchy of actions) a human is performing, including their possible future actions. GR methods aim to label a sequence of discrete observations (e.g., actions), with which (high-level) goal they belong to [27]. For instance, when provided with a sequence of move actions GR methods will attempt to select (from a predefined list) which location the agent is intending to reach. For the kitchen domain by Ramírez and Geffner [7], the sequence of observed actions includes taking different items and performing activities (e.g., making toast), and the returned classification indicates if a person is making breakfast, dinner or a packed lunch.

Methods for GR can be broadly categorised as data-driven and knowledge-driven methods [4,28]. Data-driven approaches train a recognition model from a large dataset [4,29,30,31]. The main disadvantages of this method are that often a large amount of labelled training data is required, and the produced models often only work on data similar to the training set [32,33]. Knowledge-driven approaches to GR rely on a logical description of the actions agents can perform. They can be further divided into approaches that search through a library of predefined plans (also known as “recognition as parsing” [26,27,34]) and approaches that solve a symbolic recognition problem, i.e., “recognition as planning” [2,7,27]. In our work, a graph structure is generated from a problem definition in PDDL; this graph structure is similar to those used by some recognition as parsing methods.

Recognition as parsing tends to be fast and allows multiple concurrent plans to be detected [34,35,36,37,38], but is often considered to be less flexible [7,25] because a planning library containing all actions and their orderings must be developed a priori. A planning library is usually formulated in a hierarchical structure, which includes abstract actions along with how they are decomposed to concrete (observable) actions. The link between parsing text and hierarchical plan recognition structures was suggested by [39], and since then, many plan recognition as parsing algorithms have been developed, e.g., [34,37,40].

In [41] a Temporal AND-OR tree was constructed from a library of plans to determine which objects a human will navigate to. Our method of representing a (human) agent’s actions in an Action Graph is inspired by this AND-OR tree; however, the construction of our graph is considerably different. Moreover, their AND-OR tree only contains ORDERED-AND nodes, whereas Action Graphs include both ORDERED and UNORDERED-AND nodes as often actions do not need to be performed in a fixed order.

Recognition as planning is a more recently proposed approach, in which languages normally associated with task planning, such as PDDL, define the actions agents can perform (along with their preconditions and effects) and the world state. This enables a single set of action definitions to be written for task planning, GR and GRD. Moreover, whereas in recognition as parsing usually only actions are considered, planning-based approaches allow for the inclusion of state knowledge, such as what objects are found within the environment and their locations.

Early works, such as [7], were computationally intensive as a planner was called twice for every goal, to find the difference in the cost of the plan to reach the goal with and without taking the observations into consideration. Later advances in GR as planning algorithms greatly improved the computational efficiency [2,8,9]. Plan graphs were proposed in [9] to prevent a planner from being called multiple times. A plan graph, which contains actions and propositions labelled as either true, false or unknown, is built from a planning problem and updated based on the observations. Rather than calling a planner, the graph is traversed to calculate the cost of reaching the goals. More recently, the work presented in [2,8] significantly reduced the recognition time by finding landmarks, i.e., states that must always be passed for a particular goal to be achieved.

## 4. Distinctiveness Metric

The WCD metric proposed in [10] only provides knowledge about the longest non-distinctive prefix for a set of goals G, rather than considering the overall distinctiveness of the goals and the structures of the plans. In this section, several examples are provided to illustrate the shortcomings of the WCD metric and additional metrics are proposed.

**Figure 3:**
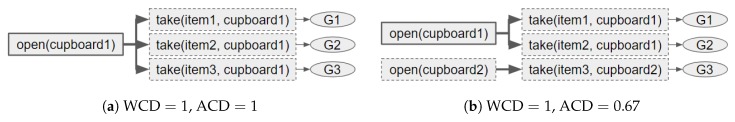
Example plans for 3 goals. The initial actions common to multiple goals (i.e., the non-distinctive prefixes) are indicated by boxes with solid borders. The remaining actions in the plans to the goals are indicated by boxes with dashed borders.

Whilst redesigning the environment, the distinctiveness of some goals ($G^{\prime }\subset G$) can be increased without affecting the WCD. We, therefore, propose finding the longest non-distinctive prefix for each goal and calculating the average length of these, namely the average distinctiveness (ACD). An example is provided to demonstrate the advantage of calculating ACD over WCD. Suppose there are three goals, all requiring a different item to be taken from the same cupboard. The plans are shown in Figure 3a, from which it is clear that the WCD of these goals is 1. After redesigning the environment, by moving item3 from cupboard1, G3 becomes fully distinctive (Figure 3b). The environment has arguably been made more distinctive but the WCD does not reflect this, i.e., it is still 1. The ACD of the initial environment is also 1, i.e., (1+1+1)/3, but after item3 has been moved, the ACD is reduced to 0.67.

A second drawback of WCD (and also of ACD) is that these metrics only capture the lengths of the non-distinctive plan prefixes and not the structure of the plans. In other words, WCD lacks knowledge of the dependencies between actions, and thus the possible plan permutations. The more dependants a non-distinctive action has, the less distinctive that action is. If an action has one distinct dependant, then only one change is required to make it fully distinctive. When the state of the environment is redesigned, both the WCD and the ACD metrics could be reduced without the goals becoming necessarily more distinctive, or vice-versa. For this reason, we introduce modified versions of WCD and ACD, i.e., ${\mathrm{WCD}}_{\mathrm{dep}}$ and ${\mathrm{ACD}}_{\mathrm{dep}}$.

To calculate these, if a non-distinctive action is required to fulfil multiple actions’ precondition(s), that action is counted multiple times. More precisely, each action *a* in the longest non-distinctive prefix is counted *C* times, with *C* equalling the number of actions (including the goal itself) for which *a* is a dependency. Dependencies are defined in this paper as actions that set one or more of the dependant’s preconditions, e.g., action 1 is said to be dependent on action 2 if action 2 fulfils one (or more) of action 1’s preconditions, i.e., $a1_{pre}\cap a2_{eff}\neq \emptyset$. If multiple options exist the longest list of dependencies is selected.

The calculation for $ACD_{dep}$ is defined by Equation (1), in which |p(G1,G2)| is the number of actions counted, as described above, in the longest prefix common to both G1 and G2. In the calculation of $WCD_{dep}$, the averaging operation is replaced by finding the maximum, see Equation (2). Note, the operator *p* is non-commutative: |p(G1,G2)| is not necessarily equal to |p(G2,G1)|. Algorithmic details on how *p* can be discovered are provided in Section 6. For clarification several examples in which $\mathrm{WCD}\neq {\mathrm{WCD}}_{\mathrm{dep}}$, and the reduction differs, are provided next.

(1) $${\mathrm{ACD}}_{\mathrm{dep}}\left(G\right)=\frac{\sum_{G1\in G}\left(\max_{G2\in (G\backslash G1)}\left|p(G1,G2)\right|\right)}{\left|G\right|}$$

(2) $${\mathrm{WCD}}_{\mathrm{dep}}\left(G\right)=\max_{G1\in G}\left(\max_{G2\in (G\backslash G1)}\left|p(G1,G2)\right|\right)$$

**Figure 4:**
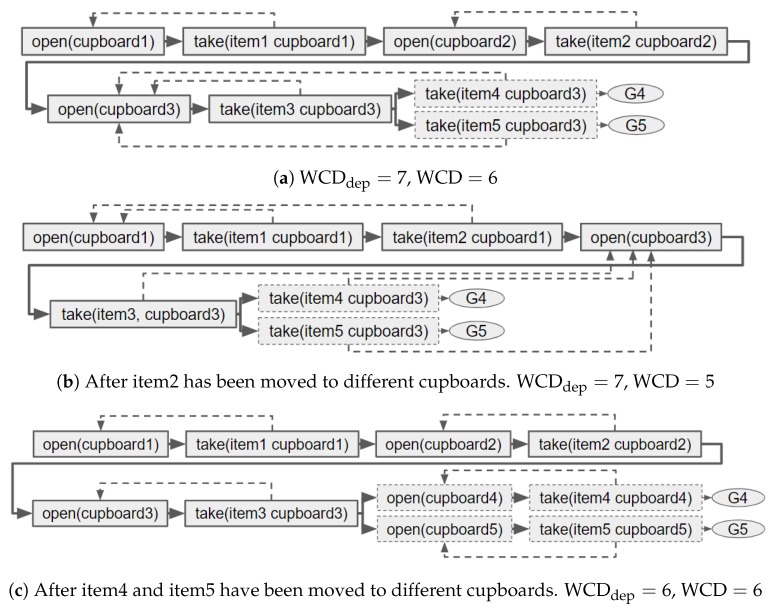
Examples in which ${\mathrm{WCD}}_{\mathrm{dep}}\neq \mathrm{WCD}$, and there reductions differ when the state of the environment is changed. A single plan permutation is provided. The dashed arrows go from dependant to dependency.

In the example shown in Figure 4a, each of the 2 goals requires taking item1, item2 and item3. These items are all in different cupboards that must be opened before the item can be taken. Therefore, the WCD for this environment is 6. The ${\mathrm{WCD}}_{\mathrm{dep}}$ is 7, as both these goals require a second (unique) item to be taken from cupboard3, so opening cupboard3 is counted twice. If during the design process item2 is moved into cupboard1 the WCD is reduced to 5 but the ${\mathrm{WCD}}_{\mathrm{dep}}$ is not reduced (Figure 4b). When items are moved into a single cupboard the plans, as well as non-distinctive prefixes, become shorter. Therefore, a GRD approach that aims to reduce WCD could simply move all items into the same cupboard. A better solution would be to put the items unique to a goal in a cupboard not required by any another goal since, depending on which plan permutation is selected, this enables the goal to be recognised after a single observation. Note, making plans shorter may be desired by the human; however, it is not the primary aim of our work.

In the same scenario, it is also possible that ${\mathrm{WCD}}_{\mathrm{dep}}$ is reduced when WCD is not. Figure 4c shows the example after such a change occurs. If the items unique to both plans are moved into different cupboards (which are also not contained within the non-distinctive prefix), ${\mathrm{WCD}}_{\mathrm{dep}}$ is reduced but WCD remains the same. This reduction reflects the fact that, due to the different plan permutations, the human’s first action could now be distinctive (i.e., if they open cupboard4 their goal must be G4).

The non-distinctive prefix p(G4,G5) does not necessary equal the non-distinctive prefix p(G5,G4). For example, if item4 is moved to cupboard4 and item5 remains in cupboard3, then |p(G4,G5)|=6, whereas |p(G5,G4)|=7 (i.e., the opening of cupboard3 is counted twice). G4 is more distinctive than G5, as to maximise G5’s distinctiveness one item’s state needs to be changed, but modifying G4’s plan will not increase the distinctiveness.

## 5. Action Graphs for Modelling GRD Problems

Goal recognition design is performed by building an Action Graph, traversing the graph to find the non-distinctive prefixes, then either removing or replacing actions to reduce the length of these non-distinctive prefixes. This section provides details on the structural features of Action Graphs and on how such an Action Graph is derived from a problem specified in PDDL.

### 5.1. Structural Features

Action Graphs model the order constraints, i.e., dependencies, between actions. An Action Graph contains OR, AND and leaf nodes. Leaf nodes are also referred to as action nodes, as each one is associated with an action. AND nodes are split into two categories: ORDERED-AND, which denotes that all children are performed in order, and UNORDERED-AND, for which all children are performed in any order. For OR nodes one child is performed. The root node is always an OR node. Throughout the paper, unless otherwise stated, the term parent(s) always refers to the direct parent(s) of a node, the same goes for child/children.

**Figure 5:**
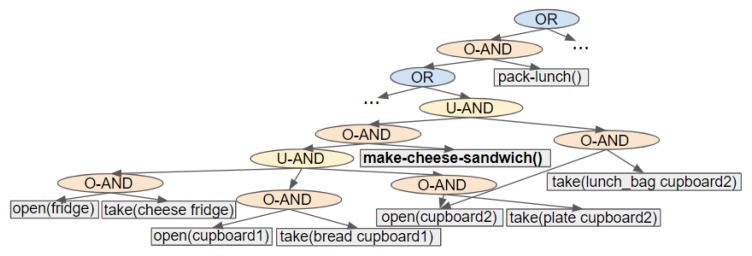
Example of an Action Graph. Arrows point towards the child node. O-AND stands for ORDERED-AND and U-AND is UNORDERED-AND. Figure only show a small sub-set of the actions in the complete kitchen domain Action Graph.

The term graph is stated, rather than tree, because action and ORDERED-AND nodes can have multiple parent nodes. Action Graphs are acyclic, i.e., do not contain any cycles. A sub-section of an example Action Graph is depicted in Figure 5.

### 5.2. Action Graph Creation

An Action Graph is generated from a GRD problem D=(F,I,A,G) by performing a Breadth First Search (BFS) backwards from each goal G∈G to the initial state *I*. This section first describes the construction of an Action Graph for unconstrained problems, i.e., problems in which all plan variations (including those longer than the optimal) are required to calculate the distinctiveness metrics. Subsequently, the optimisations made to the construction algorithm, that allow problems with constraints (i.e., navigation problems) to be handled, are detailed. Such problems have strict constraints on the order of actions and only the optimal plans are inserted into the Action Graph. The unconstrained and constrained Action Graph creation algorithms are demonstrated in the videos provided as Supplementary Materials.

#### 5.2.1. Unconstrained Action Graph Creation

In many domains, e.g., a kitchen domain, all plans (including sub-optimal plans) should be included, to calculate the distinctiveness. For example, the optimal plan for making breakfast includes making a cup of tea but there also exist longer plans, that for instance include making a cup of tea with milk or making a cup of coffee instead of tea. All these plan variations are required to calculate how distinctive making breakfast is from the alternative goals. These differences should not be inserted into the lists of hypothesis goals (G), e.g., (and (made_breakfast) (made_tea)), (and (made_breakfast) (made_coffee)), etc., for a number of reasons. First, there could be numerous different plans (e.g., in a large factory or hospital domain) to reach a goal; thus, it would be time consuming (and unnecessary) to add every plan variation as a separate goal in the list of hypothesis goals G. Second, it may be more important to determine the high-level goal of a human (e.g., are they making breakfast or dinner). GRD should make these more distinctive rather than, for instance, increasing the distinctiveness between making breakfast with tea and making breakfast with coffee. Third, in some cases this would cause it to be impossible to reduce the WCD, e.g., the WCD would always be the length of the plan to make breakfast with tea as all its actions are within the plan to make breakfast including tea with milk.

**Figure 6:**
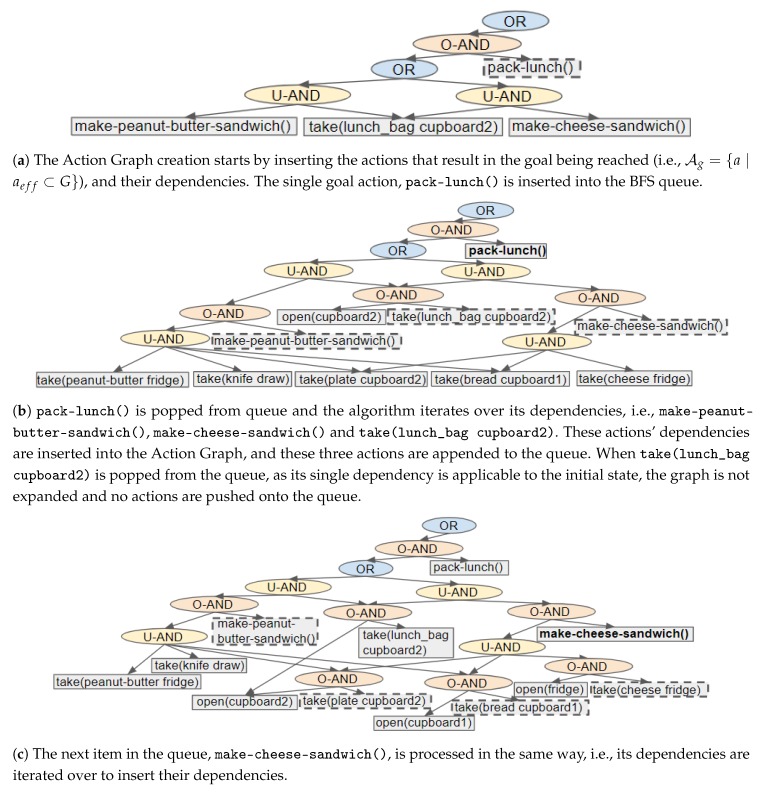
Example of the steps taken to create an Action Graph, when the first goal is (lunch_packed), which can be achieved by retrieving a lunch bag, and making either a cheese or peanut butter sandwich. Actions with a dashed border are currently in the BFS queue and the bold actions indicate the action which has just been popped from the queue. The algorithm continues until the queue is empty; only the first steps are provided to prevent the graph becoming unreadable.

Performing BFS from a goal to the initial state enables an Action Graph to be built from the root downwards. In the resulting graph, (which has a tree-like structure) the actions closest to the root result in a goal atom being met and those at the furthest points from the root are applicable in the initial state (*I*). Figure 6 shows an example of the steps executed to insert the plans for a single goal, i.e., (lunch_packed), into the graph.

An Action Graph is initialised with a OR node as the root, and each goal G∈G is processed in turn. Actions which result in a goal atom being reached ($a\in A_{g}$ with $A_{g}=\left\{a|a_{eff}\subseteq G\right\}$) are found and inserted into the graph along with their direct dependencies, as shown in Figure 6a. These goal actions are pushed onto a BFS queue to initialise it. For each action (*a*) in the queue our algorithm iterates over its dependencies, to insert the dependencies of these dependencies (e.g., Figure 6b,c into the Action Graph. During this iteration, the action’s (*a*) dependencies are pushed onto the queue. Actions and dependencies are processed in this manner because it is simpler to remove the action when none of its dependencies’ dependencies can be inserted. Dependencies are actions; therefore, in the subsequent text we refer to a dependency’s dependencies as an action’s dependencies.

To insert an action’s dependencies into an Action Graph multiple nodes are created, including an ORDERED-AND node and an action node for each dependency. If the action is a goal action, its action node is created and the ORDERED-AND node is appended to the root node’s children (e.g., Figure 6a). Otherwise, the action itself is already in the graph, and the action’s current parents become the ORDERED-AND node’s parents (e.g., Figure 6b). The ORDERED-AND node’s children are set to the dependencies followed by the action node itself. These dependencies can be a single action, a list of multiple actions or a set of alternatives (as actions can have equivalent effects, or their precondition could contain an or statement). If an action has a single dependency, a single action node is created and prepended directly to the ORDERED-AND node’s children; e.g., in Figure 6b take(lunch_bag cupboard2) has a single dependency. When an action depends on multiple actions, an UNORDERED-AND node is prepended to the ORDERED-AND node’s children. The UNORDERED-AND node’s children are set to the dependencies; for example, make-cheese-sandwich() in Figure 6b. For a set of alternatives, an OR node is inserted to indicate the different choices that can be made. In this case, the OR node is prepended to the ORDERED-AND node’s children, and the different options (i.e., actions or UNORDERED-AND nodes) become the OR node’s children (e.g., Figure 6a).

When an action that has no dependencies is reached, i.e., is applicable to the initial state ($A_{I}=\left\{a|a_{pre}\subseteq I\right\}$), there is no need to further expand that branch of the graph (i.e., the action is not pushed on to the BFS queue). Moreover, if an action was previously processed, e.g., when adding the plans for the preceding goals, there is no need to re-insert its dependencies nor to insert the action into the queue. Branches for which the initial state cannot be reached are removed. This process finishes when the BFS queue is empty.

After all actions required to reach the first goal have been inserted, the same process is repeated for the subsequent goal. The order in which the goals are processed does not affect the resulting Action Graph. To insert all plan variations, no boundary on how sub-optimal a plan can be is set. For actions (and states) that can expand abundantly, such as in navigation domains, the constrained approach described in the next section (Section 5.2.2) should be executed.

UNORDERED-AND nodes are also inserted when a goal containing an and statement, which results in more than one action being required to reach that goal, is detected. When this is detected an ORDERED-AND node is created and its children are set to a UNORDERED-AND followed by a placeholder/dummy action. The UNORDERED-AND node’s children are set to the required actions (or their parent ORDERED-AND nodes if they have dependencies). This placeholder becomes the action required to reach the goal state, i.e., $a_{eff}=G$ where *a* is the placeholder. These placeholders simplify the graph traversal algorithms required to find and reduce the non-distinctive prefixes; they are ignored when calculating the ${\mathrm{ACD}}_{\mathrm{dep}}$ and ${\mathrm{WCD}}_{\mathrm{dep}}$ metrics.

#### 5.2.2. Action Graph Creation for Navigation Problems

**Figure 7:**
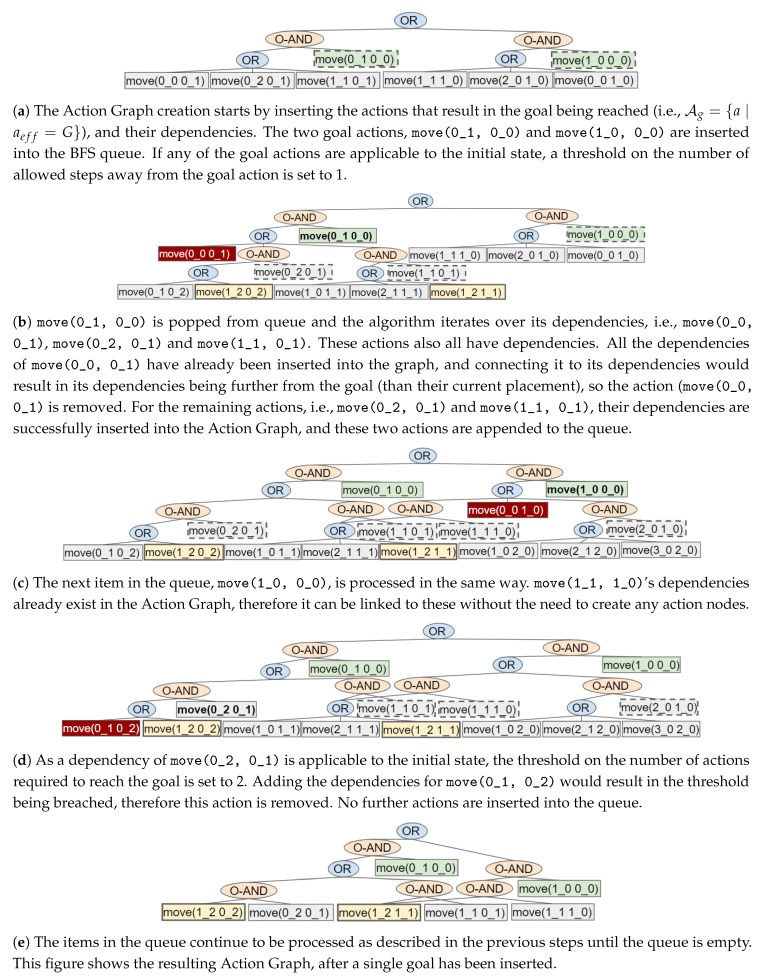
Example of the steps taken to create an Action Graph, when the initial state is (human-at 1_2) and the first goal is (human-at 0_0). The layout of the environment is shown in Figure 8. Actions which result in the goal state being reached are highlighted in green; yellow boxes with a thick border indicate actions applicable to the initial state; red indicates actions that are being removed; actions with a dashed border are currently in the BFS queue and the bold actions indicate the action which has just been popped from the queue. Reproduced from our prior work [11].

**Figure 8:**
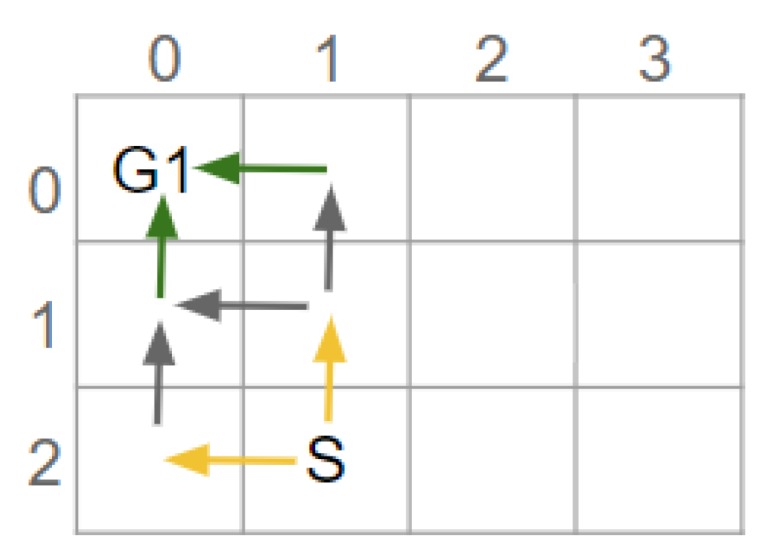
A depiction of the grid-based navigation problem, that used to describe the Action Graph creation algorithm. The arrows indicate the actions within the produced Action Graph.

For navigation problems the graph creation process can be optimised by only finding the optimal plans. This section describes how a limit is set during the Action Graph creation. The modified version of the algorithm is provided in Appendix A (Algorithm A1). For this domain, only a single permutation of a plan exists. Figure 7 shows an example of the steps executed to insert all the optimal plans for a single goal in a 3 by 4 grid-based navigation problem, as depicted in Figure 8.

Similar to before, all actions to reach a goal are inserted into the Action Graph by performing a BFS starting from actions whose effect ($a_{eff}$) result in the goal state (*G*), i.e., $A_{g}=\left\{a|a_{eff}=G\right\}$. When an action that has no dependencies, i.e., is applicable to the initial state ($A_{I}=\left\{a|a_{pre}\subseteq I\right\}$), is reached the length of the shortest plan is known; as this is the number of steps taken from the goal to reach that action (Figure 7d). Any actions not within an optimal plan, i.e., whose dependencies are further from the goal than the current limit, are removed from the graph; if there are no actions with equivalent effects, actions that are dependent on the action being removed are also removed.

Actions are also removed when linking it to its dependencies would cause a cycle (and thus sub-optimal plans) to occur. If all the dependencies of an action have been inserted into the graph while processing the current goal, then connecting it to its dependencies would create a cycle within the graph. Therefore, the action is removed. For example, the move(0_0 0_1) action in Figure 7b.

When the BFS queue is empty, all optimal plans to reach the first goal are contained within the Action Graph; an example is shown in Figure 7e. When inserting the actions that lead to the subsequent goals, the algorithm also checks if an action was inserted when processing a previous goal. If so, there is no need to (re-)insert the action’s dependencies (nor insert the action into the queue). The limit on the number of actions to reach the goal is set to the current action’s distance from the goal plus its distance from the initial state, which is discovered by traversing the graph (depth-first).

## 6. Find All Non-Distinctive Plan Prefixes

Once an Action Graph has been created, the non-distinctive plan prefixes are discovered, and the ${\mathrm{ACD}}_{\mathrm{dep}}$ and ${\mathrm{WCD}}_{\mathrm{dep}}$ are calculated. These prefixes are then iterated over to increase the distinctiveness of the goals. In our approach finding non-distinctive plan prefixes involves two steps: (1) labelling which nodes in the graph belong to which goals and (2) for each of the goals traversing the graph to find the actions that also belong to another goal’s plan (as shown in Algorithm 1). This section describes these two steps.

### 6.1. Label Which Nodes Belong to Which Goals

Each goal action $A_{g}=\left\{a|a_{eff}=G\in G\right\}$ that has dependencies, has an ORDERED-AND node as its only parent. All children of this ORDERED-AND node, including non-direct children, belong to the same goal as the goal action. Therefore, by performing a depth first traversal starting from these ORDERED-AND nodes, all nodes are labelled with which goals they belong to. This step is performed to make traversing the graph to extract the non-distinctive prefixes easier and more efficient. These labels will also be utilised by the algorithms for increasing the distinctiveness of goals.

### 6.2. Extract Non-Distinctive Prefixes

To extract the non-distinctive prefixes, goal actions are iterated over (lines 5–8 Algorithm 1) and a depth first traversal starting from each of their parent ORDERED-AND nodes is performed, to find the actions (and their dependencies) that are also in another goal’s (G2’s) plan. During this depth first traversal what steps are performed depends on the node’s type (e.g., action, OR etc.) and whether that node belongs to the second goal (G2).

- If the current node is an action node and it belongs to G2 (line 14), then it is simply appended to the list of non-distinctive actions (i.e., the prefix). Actions that do not belong to G2 are ignored.
- When the node is of type OR (line 16), each of its children are iterated over to find the one containing the most actions belonging to G2. Only one of its branches are required within a plan, and so to calculate the metrics only the longest non-distinctive prefix is required. The non-distinctive actions not within the branch containing the most actions belonging to G2, are inserted into the list of all non-distinctive prefixes, as these actions should also be processed by algorithms that attempt to minimise the ${\mathrm{ACD}}_{\mathrm{dep}}$/${\mathrm{WCD}}_{\mathrm{dep}}$.
- If an ORDERED-AND node that belongs to G2 is encountered (line 19), then all the nodes’ children (including non-direct children) also belong to G2. Therefore, a depth first traversal is performed to insert all the actions that are descendants of the ORDERED-AND node into the list of non-distinctive actions. As the left branch of an ORDERED-AND node must be performed before the right branch, actions are inserted in the order they must be performed. During this traversal, OR nodes are handled using the method mentioned above.
- In all other cases, the algorithm recurses over all the node’s children (lines 22–24).

The longest non-distinctive prefix discovered for each pair of goals (lines 5–8) is passed to Equations (1) and (2) (from Section 4) to calculate ${\mathrm{ACD}}_{\mathrm{dep}}$ and ${\mathrm{WCD}}_{\mathrm{dep}}$.

**Algorithm 1** Extract non-distinctive prefixes (depth-first traversal).  >  **Inputs**: the list of goals and the Action Graph  >  **Output**: list containing all non-distinctive plan prefixes  1:  **function**
get_non_distinctive_prefixes(G,graph)  2:   **for**
G1∈G
**do**  3:    $A_{n}=graph.get\_action\_nodes\left(a\in \left\{a^{\prime }|a_{eff}^{\prime }=G1\right\}\right)$  4:    **for**
G2∈(G\G1)
**do**  5:       **for**
$a_{n}\in A_{n}$
**do**  6:        $GET\_NON\_DISTINCTIVE\_PREFIX\_RECURSE(a_{n}.parent,G2,prefix=\left[\;\right])$  7:        allPrefixes.append(prefix)  8:       **end for**  9:    **end for** 10:   **end for** 11:   sort(allPrefixes)▹ longest first 12:  **end function** 13:  **function**
get_non_distinctive_prefix_recurse(node, G2, prefix) 14:   **if** node is action node **and**
node.belongsTo(G2)
**then** 15:    prefix.append(node) 16:   **else if** node is OR node **then** ▹ Get longest non-distinctive prefix, all others are appended to allPrefixes 17:    longestPrefix=get_longest_non_distictive_prefix(node.children,G2) 18:    prefix.append_all(longestPrefix) 19:   **else if** node is ORDERED-AND
**and**
node.belongsTo(G2)
**then** 20:    prefix.append_all(node.get_all_leaves_dfs()) 21:   **else** 22:    **for**
child∈node.children
**do** 23:       GET_NON_DISTINCTIVE_PREFIX_RECURSE(child,G2,prefix) 24:    **end for** 25:   **end if** 26:  **end function**

## 7. Performing Action Replacement to Reduce ACD

Modifications applied to the world state *I* can cause actions in a plan to be replaced by more distinctive actions, and thus reduce ${\mathrm{ACD}}_{\mathrm{dep}}$. For example (as shown in Figure 3), if the state of the environment is modified by moving item3 from cupboard1 to cupboard2, in all plans opening cupboard1 then taking item3 is replaced by opening cupboard2 then taking item3. In our approach, part of the Action Graph is replaced by another sub-graph. This section describes how the list of actions and their replacements is formulated, followed by two methods for selecting which actions to replace, i.e., exhaustive and our less computationally expensive method Shrink–Reduce.

### 7.1. Defining Modifications

This section describes how the possible modifications are defined; the process that creates the replacement sub-graphs; and how these modifications are applied to the Action Graph by the exhaustive and Shrink–Reduce algorithms. The modifications applied during the environment design process are generated from additional PDDL action definitions. For instance, the move-item-state-modification(?item ?container1 ?container2) action definition, along with its preconditions and effects, is filled in with all combinations of items and containers that are stated in a PDDL defined problem. The modifications (actions) not applicable to the initial state are filtered out. An example definition is provided in Appendix B.

**Figure 9:**
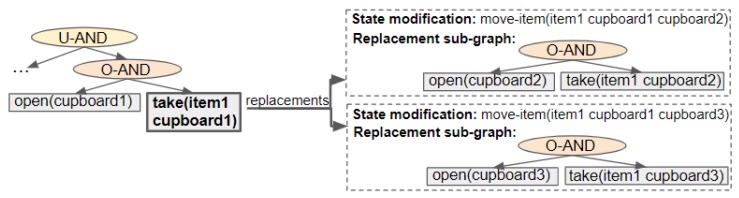
Example of an action currently in the Action Graph, i.e., take(item1 cupboard1), which can be affected by 2 state modification actions. Thus, it can be replaced, by swapping its parent ORDERED-AND node with one of its replacement sub-graphs.

Our action replacement algorithms start by creating a map, that maps action nodes to their replacement graphs. For each modification, a list of actions affected by the modification is extracted. An action is affected by a modification if its preconditions ($a_{pre}$) contain a variable of which the value is different in the modification’s effects ($m_{eff}$). Subsequently, a replacement sub-graph is generated by first applying a modification to the initial state *I*, then creating a (separate) Action Graph for a goal set to an affected action’s effects ($a_{eff}$). The resulting map contains a list of actions along with their replacement sub-graphs and corresponding modifications. An example of this is visualised in Figure 9.

Changes are made to the graph by swapping the action node(s) (or, if the action has dependencies, its parent ORDERED-AND node) with the appropriate sub-graph(s). If a replacement is selected and the corresponding modification affects other actions, they will also be replaced with their equivalent replacements. Actions are not repeated within the Action Graph; thus, nodes’ parents and children are altered if an action in the replacement already exists (rather than creating a new action node).

### 7.2. Exhaustive

The exhaustive approach iteratively applies each modification to the Action Graph, then pairs of modifications, then triplets, etc. After a set of action nodes in the Action Graph has been replaced with their corresponding sub-graphs, the ${\mathrm{ACD}}_{\mathrm{dep}}$ is re-calculated. The set of modifications resulting in the lowest ${\mathrm{ACD}}_{\mathrm{dep}}$ is returned. These modifications can be applied to *I* to produce the resulting environment design. While this algorithm is guaranteed to find the best possible solution, its computational time can become exceedingly high, especially for problems with large state spaces. Therefore, a maximum size for the set of modifications (*N*) is provided as a parameter. The pseudo-code for this process is provided in Appendix C.

### 7.3. Shrink–Reduce

Our Shrink–Reduce heuristic is a two step process: (1) Shrink all plans and (2) reduce the length of the non-distinctive prefixes. The pseudo-code can be found in Algorithms 2 and 3. A video showing the Shrink–Reduce process, for an example problem, has been provided as Supplementary Materials. In this section their details are described in turn, then an example is provided. This method has been developed as it is less computationally expensive than an exhaustive search; however, it is not guaranteed to find the solution with the lowest ${\mathrm{ACD}}_{\mathrm{dep}}$. Moreover, our Shrink–Reduce method was designed for problems containing plans with multiple permutations.

#### 7.3.1. Shrink Plans

Shrinking the plans makes it possible to increase the distinctiveness of the environment by performing a single replacement, as afterwards there will exist unused actions that can replace parts of the non-distinctive prefixes. In other words, if there are *x* variables (e.g., items) that can have *y* different values (e.g., locations), after changing all variables (that can change value) to 1 value, there are some (e.g., |y|−1) unused values that can be utilised to increase the distinctiveness. Without this step it is more difficult to reduce the non-distinctive prefixes, as a single modification often does not affect ${\mathrm{ACD}}_{\mathrm{dep}}$. The process described in this section is performed on each goal G∈G in turn.

All actions in the plans to reach a goal are extracted from the graph (Algorithm 2 line 3) by performing a depth-first search starting from the goal action’s ($a_{eff}=G$) parent (ORDERED-AND node). Actions inserted when processing a preceding goal are discovered (i.e., $A_{r}$ from line 4), to prevent increasing the length of a previously shortened plan. Subsequently, all the actions in the plans that can be replaced are iterated over (line 5). If replacing an action results in the plan being shortened (line 7), the replacement is applied to the Action Graph (line 8) and all actions in the replacement’s sub-graph are appended to $A_{r}$ (line 9); otherwise the original action (and its dependencies including indirect dependencies) are appended to $A_{r}$ (line 14). This check is performed by comparing the number of elements in the set containing $A_{r}$ plus the original action and its dependencies (including dependencies’ dependencies), with the number of elements in the set containing $A_{r}$ plus the replacement’s actions. During this check, repeated actions are ignored. Performing this comparison with $A_{r}$, rather than the full plan, increases the amount the plans are shrunk (e.g., if item1 has already been moved to cupboard2, all items should be moved to cupboard2; rather than another cupboard that appears elsewhere in the plan). This process is likely to increase the ${\mathrm{ACD}}_{\mathrm{dep}}$; however, in the next step it will be reduced.

**Algorithm 2** Shrink plans.  >  **Inputs**: list of goals, the Action Graph and map of action node to their possible replacements (e.g., Figure 9)  >  **Output**: Action Graph containing the shrank plans.  1:  **function**
shrink_plans(G,graph,actionsAndReplacements)  2:   **for**
G∈G
**do**  3:     allPlans=graph.get_all_plans(G)  4:     $A_{r}=get\_already\_replaced\_actions\left(allPlans\right)$  5:     **for**
$a\in \{a^{\prime }|a^{\prime }\in allPlans,a^{\prime }\in actionsAndReplacements.keys\}$
**do**  6:      **for**
r∈actionsAndReplacements[a]
**do**  7:       **if**
$|set\left(A_{r},r.actions\right)|<|set\left(A_{r},get\_preceding\_actions\left(a\right)\right)|$
**then**  8:          graph.replace_action(a,r)  9:          $A_{r}.append\_all\left(r.actions\right)$ 10:          **break** 11:       **end if** 12:      **end for** 13:      **if** no r∈actionsAndAlternatives[a] has been appended to $A_{r}$
**then** 14:       $A_{r}.append\_all\left(get\_preceding\_actions\left(a\right)\right)$ 15:      **end if** 16:     **end for** 17:   **end for** 18:  **end function**

#### 7.3.2. Reduce Non-Distinctive Prefixes

The second step attempts to reduce the length of the longest non-distinctive prefix for each pair of goals (e.g., p(G1,G2) and p(G2,G1)). For each prefix, this step iterates over the replaceable actions (line 5 of Algorithm 3); a replaceable action appears anywhere in the remainder of the plan (that the prefix belongs to), provided that at least one of its dependencies is within the prefix and the action itself is not in the prefix. An action is replaced by each of its possible replacements in turn (line 7), until the prefix has been shortened and the ${\mathrm{ACD}}_{\mathrm{dep}}$ reduced (lines 11–13), or all its replacements have been processed. If a replacement does not shorten the prefix and reduce ${\mathrm{ACD}}_{\mathrm{dep}}$ the alteration is undone (line 15). During this process the currently applied modifications are tracked and, once the non-distinctive prefixes have been processed, they are returned (line 21). This list of modifications (i.e., actions) can be applied to the initial state *I* to get the resulting environment design.

**Algorithm 3** Reduce non-distinctive prefixes  >  **Inputs**: list of goals, the Action Graph and map of action node to their possible replacements (e.g., Figure 9)  >  **Output**: list of modifications (actions) to produce the resulting environment design  1:  **function**
reduce_non_distinctive_prefixes(G,graph,actionsAndReplacements)  2:   prefixes=GET_NON_DISTINCTIVE_PREFIXES(G,graph)  3:   lowestAcd=calculate_acd()  4:   **for**
p∈prefixes
**do**  5:     **for**
replaceableAction∈getReplaceableActionsThatAffectThePrefix(actionsAndReplacements,p)
**do**  6:      **for**
r∈actionsAndReplacements[replaceableAction]
**do**▹ Iterate over an action’s replacements  7:       graph.replace_action(a,r)  8:       allPrefixes=GET_NON_DISTINCTIVE_PREFIXES(G,graph)  9:       $p^{\prime }=allPrefixes.get\_equivalent\left(p\right)$▹ i.e., whose goals match 10:       acd=calculate_acd() 11:       **if**
$|p^{\prime }|<|p|\;\mathrm{and}\;acd<lowestAcd$
**then**  12:          lowestAcd=acd  13:          break 14:       **else**  15:          graph.undo_replacement(a,r) 16:       **end if** 17:      **end for** 18:      prefixes.remove_equivalent(p)▹ i.e., whose goals match 19:     **end for** 20:   **end for** 21:   **return**
graph.extract_modifications() 22:  **end function**

#### 7.3.3. Example of Performing Shrink–Reduce

**Figure 10:**
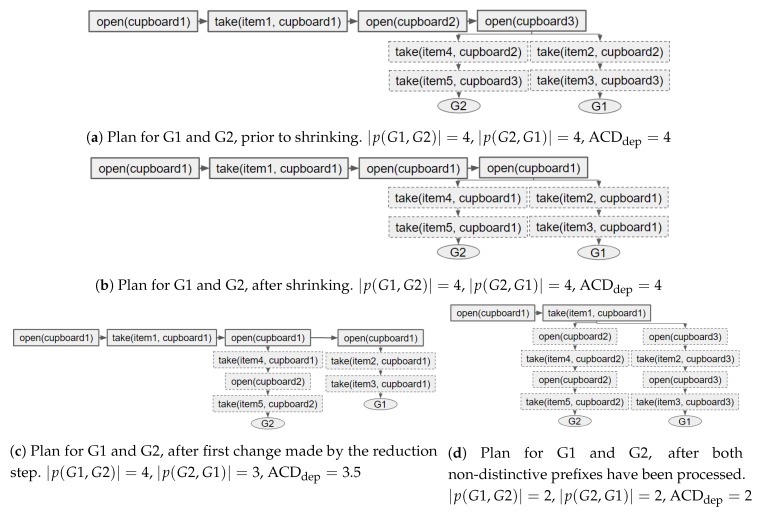
Example of shrinking the plans, then reducing the non-distinctive prefix. Only a single plan permutation is displayed. Rather than showing arrows to indicate when a non-distinctive action is counted multiple times to calculate |p(G1,G2)| and |p(G2,G1)| (as the dependency is required by multiple actions), these actions (dependencies) are repeated.

To clarify why this two step process is performed an example is provided, and depicted in Figure 10. In this example, there are 2 goals (G1 and G2) and to reach these goals different items must be taken from cupboards. The state of the environment can be altered by changing the locations of items. Prior to redesigning the environment, all available cupboards must be opened to reach either goal (Figure 10a). Therefore, it is impossible to alter the ${\mathrm{ACD}}_{\mathrm{dep}}$ (i.e., the length of the non-distinctive prefixes) with only a single modification. After shrinking the plans, i.e., moving all items to single cupboard (Figure 10b), a single modification can be applied to reduce ${\mathrm{ACD}}_{\mathrm{dep}}$; for instance, as shown in Figure 10c, item5 can be moved to cupboard2. The resulting plans and non-distinctive prefixes, after all actions in both non-distinctive prefixes (i.e., p(G1,G2) and p(G2,G1)) have been reduced, are depicted in Figure 10d.

## 8. Performing Action Removal to Reduce ACD

**Figure 11:**
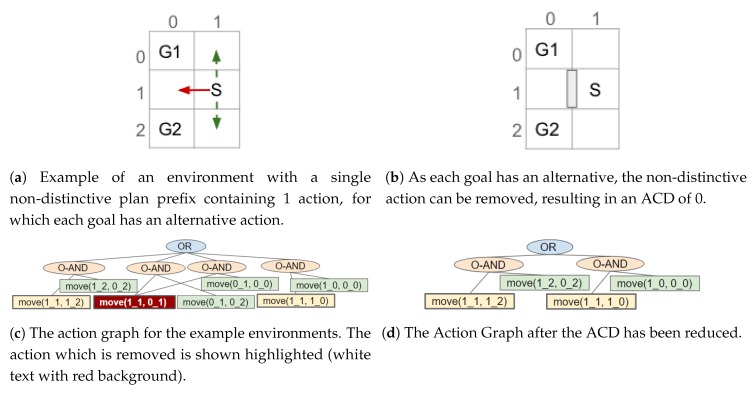
Example GRD problem, in which both goals have an alternative to the plan(s) containing the non-distinctive prefix. In all example environments a longest non-distinctive plan is indicated with red arrows, and the alternative action(s) by a green dashed arrow.

**Figure 12:**
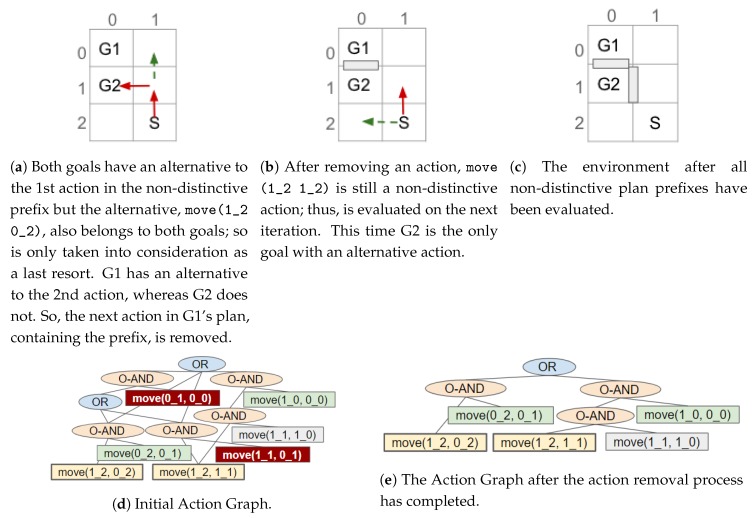
Example of ACD reduction, when only a subset of the goals have an alternative action, to an action in the worst non-distinctive plan prefix.

**Figure 13:**
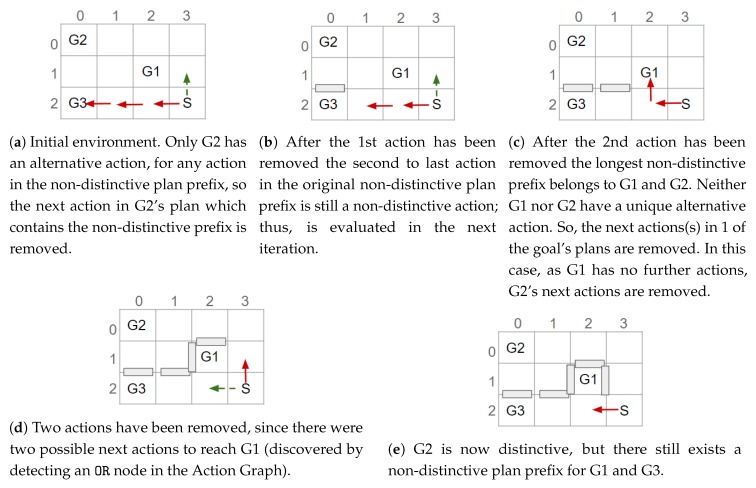
Example of a 3 goal environment, in which WCD (and thus ACD) cannot be reduced to 0.

As described in our previous work [11], by removing the possibility of performing an action and, consequently, modifying the state of the environment, the goals G can be made more distinctive (i.e., the ${\mathrm{ACD}}_{\mathrm{dep}}$ reduced). This type of state modification is applied to a navigation domain, as it is the only human-inhabited environment, we can think of, in which it is feasible to remove actions (e.g., by placing obstacles). As mentioned above, for such a domain only the optimal plans are contained within the Action Graph; thus, during the action removal process the cost of the optimal is not increased. Rather than simply exhaustively removing parts of the graph, a much less computationally expensive algorithm has been developed. This section first provides an overview of how the non-distinctive prefixes are processed, before providing the details. In Figure 11, Figure 12 and Figure 13, taken from our previous paper [11], some simple examples are provided to illustrate how our approach works. An example is also provided in a video supplied as Supplementary Materials. Our approach is applicable to environments of any size with any number of goals.

A video showing the Shrink–Reduce process, for an example problem, has been provided as Supplementary Materials. The unconstrained and constrained Action Graph creation algorithms are demonstrated in the videos provided as Supplementary Materials.

The list of non-distinctive plan prefixes is sorted, most costly first, so that the worst is processed first. In turn each prefix is taken from the list and its actions iterated over to discover if: (1) all goals an action belongs to have a (unique) alternative action; (2) a sub-set of the goals the non-distinctive prefix belongs to have an alternative; or (3) all the non-distinctive prefix’s goals have an action with an non-unique alternative. These points are expanded on below. The actions are iterated over in order because if an action at the start of the prefix can be removed, the remainder of the prefix is no-longer valid. If the algorithm were to start from the last action, in a plan containing the non-distinctive prefix, it would be more difficult to reduce the distinctiveness (i.e., more actions would require removing).

A goal has an alternative action if an action (or if the action has dependencies its single ORDERED-AND parent) has an OR node as a direct parent and another of the OR node’s children belong to that goal. If so, an action can be removed without causing the goal to become unreachable. Moreover, the alternative cannot belong to any of the other goals the (non-distinctive) action belongs to as removing this action would have no effect on the goals’ distinctiveness. If all the goals with a plan containing the non-distinctive action have an alternative, the action is removed. An example is provided in Figure 11.

After checking all actions in a non-distinctive prefix, if only a subset of the goals have alternative actions, the action(s) directly after the non-distinctive prefix in their plans is removed. As a result, those goals can only be reached by their alternative (possibly more distinctive) plan(s). An example is shown in Figure 12. Otherwise, when all the non-distinctive prefix’s goals have an action (in the non-distinctive prefix) with an non-unique alternative, i.e., the alternative(s) belongs to more than one of the action’s goals, the next (distinctive) action(s) for one of the goals is removed (see Figure 13c,d). This prevents the non-distinctive prefix being valid for that goal, thus making it more distinctive. Our action removal method always checks that the action removed will not interfere with any of the other goals, which do not have an alternative, to prevent them from becoming unreachable.

Once an action has been removed, which nodes belong to which goal is re-evaluated (see Section 6); and the actions in the non-distinctive prefix, prior to any removed action, are checked to see if they should be inserted into the list of non-distinctive plan prefixes (e.g., Figure 12a,b). Actions, that are not the last action in the prefix, should be re-processed as it may be possible to further reduce the length of the non-distinctive prefix (e.g., Figure 13a–c). The last action in the prefix will not be processed again, if it is still a non-distinctive action then the ACD will not be reduced to 0. An example of an environment in which the ACD cannot be reduced to 0, and the steps our algorithm performs, is shown in Figure 13.

## 9. Experiments

Through experiments we aim to demonstrate the scalability and performance of our goal recognition design approach. First, Shrink–Reduce is compared to the exhaustive search on problems generated from a scalability testing domain we developed. Second, the results of running Shrink–Reduce and exhaustive on a kitchen domain are presented. Finally, our action removal method is compared to the pruned-reduce method by Keren et al. [10], on grid-based navigation problems of various sizes.

### 9.1. Action Replacement Scalability Experiments

The purpose of experiments in this section is to demonstrate the performance of our Shrink–Reduce method, and compare this to the exhaustive approach. The results show the computation time, ${\mathrm{ACD}}_{\mathrm{dep}}$ reduction, ${\mathrm{WCD}}_{\mathrm{dep}}$ reduction and number of states modifications required to get from the provided to the designed world model. As the plans’ lengths can be altered during this process, the average change in length is also mentioned. The experiment setup is described, before presenting and discussing the results.

#### 9.1.1. Setup

For these experiments a test domain was developed, that contains definitions for the following actions: open(?container), take(?item ?container) and multiple goal actions, e.g., goal1(). Each goal action was generated by randomly selecting between 3 to 10 items that require taking before the goal action can be performed, i.e., its preconditions are set to a list of (taken ?item) fluents, and its effects were set to a single fluent e.g., (goal1_reached). These actions definitions are representative of definitions suitable for many application domains, e.g., a kitchen, factory or hospital. The location of each item was also selected randomly. The location of these items can be modified during the environment design process, for example, if open(cupboard1) and take(item1 cupboard1) are in a plan they can be replaced by open(cupboard2) and take(item1 cupboard2).

Three datasets were created from this test domain, (1) with a differing number of variables (i.e., items) whose value can modified, (2) an increasing number of values the variables can be set to (i.e., cupboards the items can be in), and (3) a varying number of goals. When one of these amounts is changed the others are fixed, i.e., there are 15 variables, 5 values, and 5 goals. As the datasets contain an element of randomness, for each variation 5 problems were created and the average result is presented. Experiments were ran on a server with 11 GB of RAM and a Intel Xeon CPU 2.27 Ghz processor. All graph’s error bars show the minimum and maximum result.

The exhaustive approach was ran for varying values for the maximum number of changes that can be applied (*N*), i.e., 1, 2, 3 and 4. In the results these are named *exhaustive1*, *exhaustive2*, *exhaustive3* and *exhaustive4* respectively. At N=5, the exhaustive search can take 2 days to complete a single (relatively large) problem. Therefore, for the scalability experiments *N* was set to a maximum of 4. For the experiment presented in the subsequent section (Section 9.2), as a domain with fewer goals and possible modifications was used, no limit was set for *N*.

The ${\mathrm{ACD}}_{\mathrm{dep}}$ and ${\mathrm{WCD}}_{\mathrm{dep}}$ reductions are presented in performance profile graphs, as suggested by Dolan and Moré [42]. This enables the results to be presented in a more readable format, and all datasets can be grouped into a single result, to prevent a small number of problems dominating the discussion. For run-times, graphs showing the individual data-points are provided, so the exponential increase in run-time when the problem size is increased can be clearly seen. To produce the performance profile of an approach (S∈S) the ratio between its result ($D_{P,S}$) and the best solution for a problem (P∈P) is calculated, as shown in Equation (3). Equation (4) calculates the percentage of problems an approach solved when the ratio is less than a given threshold, τ. When the ${\mathrm{ACD}}_{\mathrm{dep}}$/${\mathrm{WCD}}_{\mathrm{dep}}$ reduction is 0 ($D_{P,S}=0$), the ratio is set to infinity (as the approach failed to provide a solution). As approaches can produce the same reduction, the sum of all approaches’ performance at τ=1 does not necessarily equal 0.

(3) $$R_{P,S}=\left\{\begin{array}{cc} \frac{\max\left(D_{P,S}:S\in S\right)}{D_{P,S}} & D_{P,S}\neq 0\\ \infty & otherwise \end{array}\right.$$

(4) $$P_{S}\left(\tau \right)=\frac{1}{\left|P\right|}\left|P\in P:R_{P,S}\leq \tau \right|$$

#### 9.1.2. Results and Discussion

**Figure 14:**
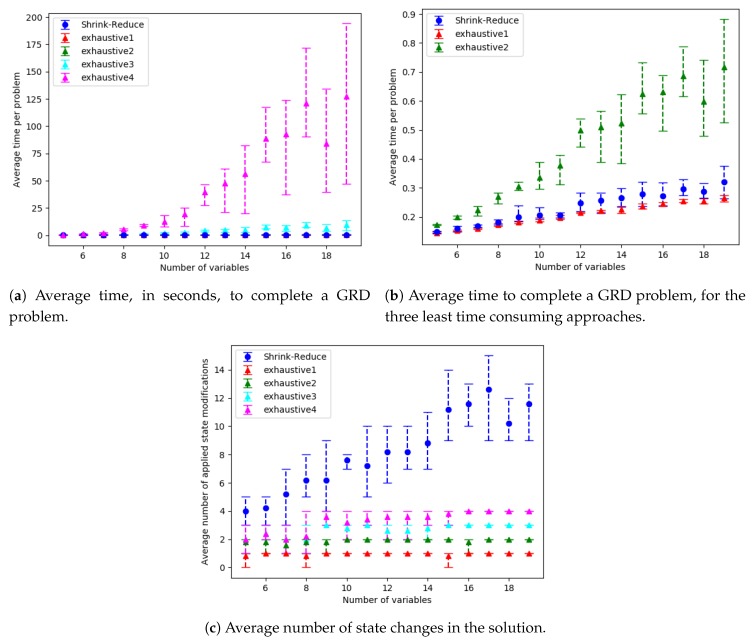
Results for an increasing number of variables.

**Figure 15:**
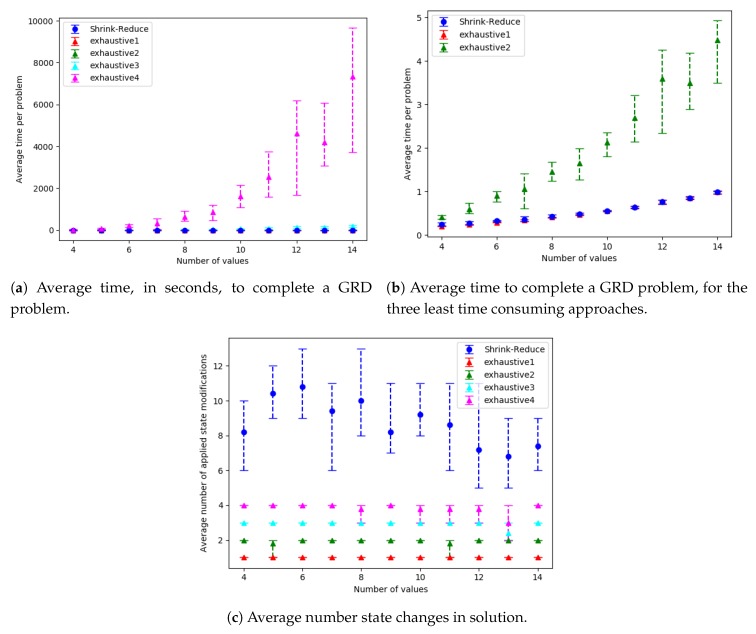
Results for an increasing number of values.

**Figure 16:**
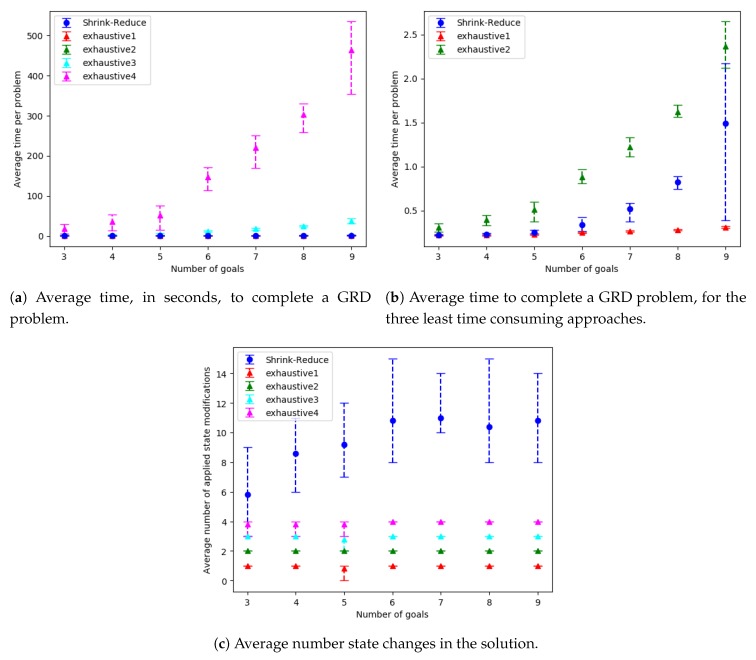
Results for an increasing number of goals.

**Figure 17:**
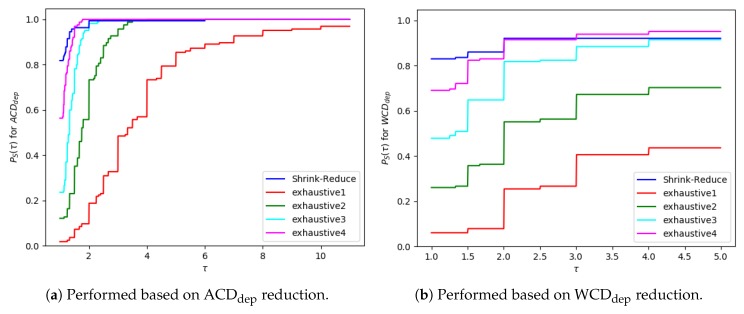
Results for all action replacement experiments. When τ is further increased, there is no change in $P_{s}\left(\tau \right)$; unless τ=∞, then $P_{S}\left(\tau \right)=1$ for all solutions.

The run-time and number of required state changes, for an increasing number of variables, values and goals are shown in Figure 14, Figure 15 and Figure 16, respectively; the ${\mathrm{ACD}}_{\mathrm{dep}}$ and ${\mathrm{WCD}}_{\mathrm{dep}}$ reduction comparisons are shown in Figure 17 (separate results for each dataset are provided in Appendix E.1). A similar trend was observed in each of these configurations. The computational times for the different approaches, followed by the increase in goal distinctiveness, are discussed.

As the size of the problem (i.e., number goals, values or variables) was increased, there was an exponential increase in the average time taken by each of the approaches. The outliers of this trend (e.g., in Figure 14a at 18 variables) are due to the nature of generating data with randomness, e.g., if a variable is not within a plan to any of the goals, the actions associated with that variable are not inserted into the graph, and thus not processed when increasing the distinctiveness of goals. Our Shrink–Reduce method finished in less time than exhaustive, except for exhaustive1 (i.e., N=1). Having said that, exhaustive1 was inferior at increasing the distinctiveness of the goals.

**Figure A3:**
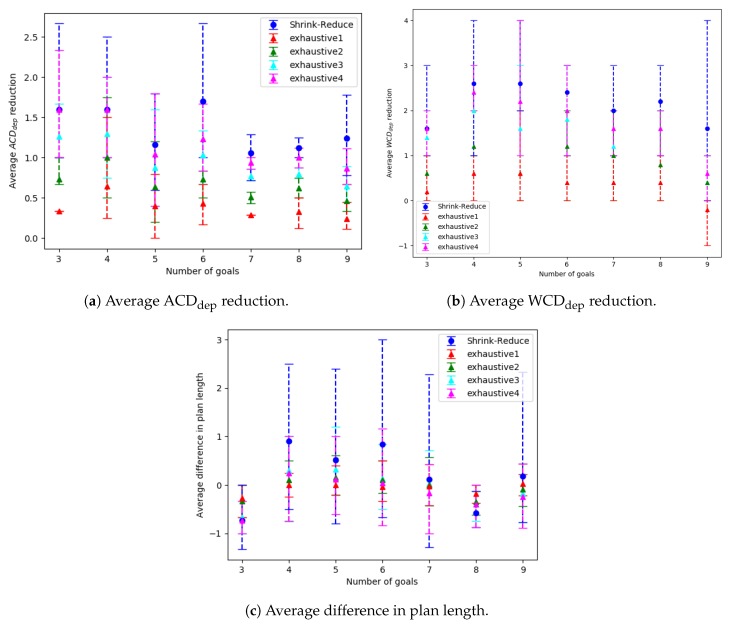
Results for an increasing number of goals.

For exhaustive, higher values of *N* allow ${\mathrm{ACD}}_{\mathrm{dep}}$ (Figure 17a) and ${\mathrm{WCD}}_{\mathrm{dep}}$ (Figure 17b) to be reduced to lower values. If exhaustive performs all possible combinations of state changes, it is guaranteed to find the best environment design. Nevertheless, our Shrink–Reduce approach often made the goals more distinctive than exhaustive4, but it also performed a large number of state changes (Figure 14c, Figure 15c and Figure 16c) and often (slightly) increased the average plan length (Appendix E.1). As stated earlier, and reiterated by these results, ${\mathrm{ACD}}_{\mathrm{dep}}$ is sometimes reduced without any reduction in ${\mathrm{WCD}}_{\mathrm{dep}}$; moreover, it is possible that ${\mathrm{WCD}}_{\mathrm{dep}}$ is increased when ${\mathrm{ACD}}_{\mathrm{dep}}$ is decreased (e.g., exhaustive1 Figure A3b). At τ≥2, the percentage of problems solved by exhaustive4 is higher than Shrink–Reduce; showing that on the problems Shrink–Reduce did not have the highest ${\mathrm{ACD}}_{\mathrm{dep}}$ reduction it is further from the best approach, than exhaustive4 is when it did not produce the best result.

With some alternative GRD methods, such as [10], it would be more difficult to calculate ${\mathrm{ACD}}_{\mathrm{dep}}$ and ${\mathrm{WCD}}_{\mathrm{dep}}$. In [10], a task planner is called to find a plan to reach a pair of goals, this joint plan contains the longest non-distinctive prefix. Our distinctiveness metrics could be calculated by searching joint plans for actions that have dependencies (i.e., should be counted multiple times); however, this would be computationally expensive and, as multiple prefixes could be of equal size, multiple joint plans (for a single pair of goals) may require processing. Methods that create graph or tree structures, such as MDPs [12] or planning libraries [21] could employ a similar method to Action Graphs. Wayllace et al. [12] perform stochastic GRD with MDPs, that each contain a single terminal (goal) state; these MDPs can be traversed to find the non-distinctive prefixes, include those actions that should be counted multiple times. In future work, we intend to investigate stochastic GRD in more detail.

### 9.2. Action Replacement Applied to a Kitchen Domain

The kitchen domain contains the actions and world model required for a human to make several meals, i.e., breakfast, a packed lunch and dinner. Ramírez and Geffner [7] created the original version of the kitchen domain for their work on goal recognition, based on the work by Wu et al. [16]. Moreover, similar problems have been utilised by several related works, e.g., the cooking problem by Yordanova et al. [4]. This experiment is included to demonstrate our approach applied to a realistic domain containing more action definitions (than our scalability testing domain). Rather than just a goal action preceded by a list of take item actions, a more complex graph (plan) structure was produced. Further, fewer goals, variables and values were contained within the kitchen goal recognition design problem, therefore running exhaustive to find the best possible solution was feasible.

#### 9.2.1. Setup

We extended the original kitchen domain by Ramírez and Geffner [7] with action definitions and the state knowledge required to take items from cupboards, a fridge and a drawer; the newly defined actions are open(?container), close(?container) and take(?item ?container). The initial state of the environment is set so that all food items (that don’t need to be stored in the fridge) are in cupboard1, equipment (e.g., cup and plate) is in cupboard2 and all utensils (e.g., spoon and knife) are in the drawer. In this dataset there are 3 hypothesis goals: G={(made_breakfast), (lunch_packed) and (made_dinner)}. These goals require multiple items to be taken and other actions to be performed, e.g., part of the plan to make breakfast involves taking bread and making toast. Each goal can be achievable through multiple plans, e.g., for (lunch_packed) to be reached a person must always perform the take(lunch_bag) action but has the option of either performing the make-peanut-butter-sandwich() or make-cheese-sandwich() action. Information on the plans producible from this domain are provided in Appendix D.

Like the previous experiment, the location of items is modified during the environment design process. As it is likely that a human would not want items within the fridge or drawer to be changed (e.g., milk taken out the fridge, or cereal put in the drawer), PDDL not statements have been inserted into the state modification action to prevent this. The PDDL definition for this action is provided in Appendix B.

#### 9.2.2. Results and Discussion

For the kitchen domain, the initial ${\mathrm{WCD}}_{\mathrm{dep}}=14$ and ${\mathrm{ACD}}_{\mathrm{dep}}=11.00$. Both our Shrink–Reduce and exhaustive approaches successfully increase the distinctiveness of the goals. Exhaustive (although slower) made the goals more distinctive than Shrink–Reduce. Shrink–Reduce reduced ${\mathrm{WCD}}_{\mathrm{dep}}$ to 12 and ${\mathrm{ACD}}_{\mathrm{dep}}$ to 10.00, whereas exhaustive reduced ${\mathrm{WCD}}_{\mathrm{dep}}$ to 11 and ${\mathrm{ACD}}_{\mathrm{dep}}$ to 9.33. This illustrates that, for environments with fewer state changes, the exhaustive algorithm should be executed.

Both approaches reduced the WCD (distinctiveness metric by Keren et al. [10]) by 1 action. ${\mathrm{WCD}}_{\mathrm{dep}}$ was lowered more than WCD as the number of repeated open actions (that are dependencies for take actions) was also reduced (as explained in Section 4). This difference indicates that depending on which plan permutation is selected by a human, their goal can now be determined sooner. As demonstrated by these results, for problems with multiple plan permutations, ${\mathrm{WCD}}_{\mathrm{dep}}$ can provide more insight into the distinctiveness of an environment than just providing WCD.

To produce the resulting environment designs of either approach, 3 state modifications need to be applied to the initial state (*I*). The list of required modifications are provided in Table 1. Both approaches proposed moving bread from cupboard1 to cupboard2, this results in cupboard1 only needing to be opened when making breakfast, and not when making dinner or a pack lunch. Therefore, if a human opens cupboard1 their goal is known. Moving the water-jug and/or cup to cupboard1 decreases ${\mathrm{ACD}}_{\mathrm{dep}}$ as these items are only required to make breakfast. Shrink–Reduce moved the bowl instead the cup into cupboard1. This increased the distinctiveness between making dinner (which requires the bowl) and making a pack lunch (which does not require the bowl). After moving the bowl, changing the cup’s location would not have altered the distinctiveness (as cupboard1 requires opening for both making breakfast and dinner).

During some initial tests we enable items to be moved into/from the fridge and drawer. This resulted in a lower ${\mathrm{ACD}}_{\mathrm{dep}}$ (than provided in Table 1) for the redesigned environment produced by both methods. Nonetheless, some changes, e.g., moving the bread into a drawer, are undesired by humans. In many scenarios there will be a trade-off between making undesired changes and increasing the goals’ distinctiveness.

### 9.3. Action Removal Experiments

The action removal experiments, presented in this section, aim to show: (1) How well our approach scales as the number of goals and grid size of a navigation domain are increased, (2) how much the ACD and WCD is reduced, and (3) how many actions are removed. For this domain $\mathrm{ACD}={\mathrm{ACD}}_{\mathrm{dep}}$ and $\mathrm{WCD}={\mathrm{WCD}}_{\mathrm{dep}}$, as actions are strictly ordered. Our Action Graph approach is compared to the pruned-reduce method by Keren et al. [10], by running both approaches on a dataset we generated, containing problems with randomly selected initial and goal locations. This section first describes the experiment setup, then discusses the results.

#### 9.3.1. Setup

Two grid-based navigation datasets containing GRD problems were created. The first contains problems with an 8 by 8 grid and a varying number of goals (destinations). For each number of goals, 8 problems were generated by randomly selecting a start location and the goals’ locations. In total the dataset contains 112 problems.

The second dataset consists of problems with differing grid sizes, for all problems both the width and the height are equal. For each grid size 8 problems, with a random start location and three random goal locations, were generated. In total this dataset contains 56 GRD problems.

Experiments were ran on our department’s server with 3 GB of RAM and a Dual Core AMD Opteron 2 Ghz processor. A timeout of 10 min per GRD problem was set for all experiments. The whole process, including converting the PDDL into an Action Graph is included in the run-times for our approach. The output of pruned-reduce does not state the ACD (as this is our new metric), so to calculate this the resulting environment design is passed to our algorithm’s ACD calculation. As before, all graph’s error bars show the minimum and maximum result.

#### 9.3.2. Results and Discussion

**Figure 18:**
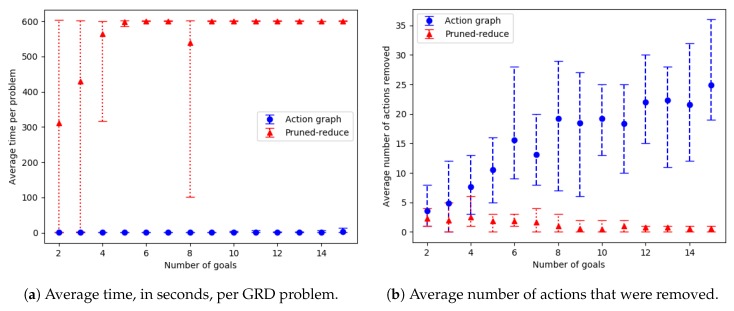
Results for an increasing number of goals. The results of our Action Graph approach are indicated by blue circles, pruned reduce [10] is shown with red triangles. All times are given in seconds.

**Figure 19:**
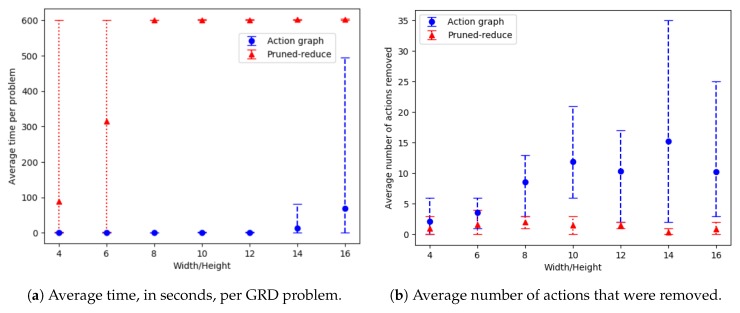
Results for an increasing grid size. The results of our Action Graph approach are indicated by blue circles, pruned-reduce [10] is shown with red triangles. All times are given in seconds.

**Figure 20:**
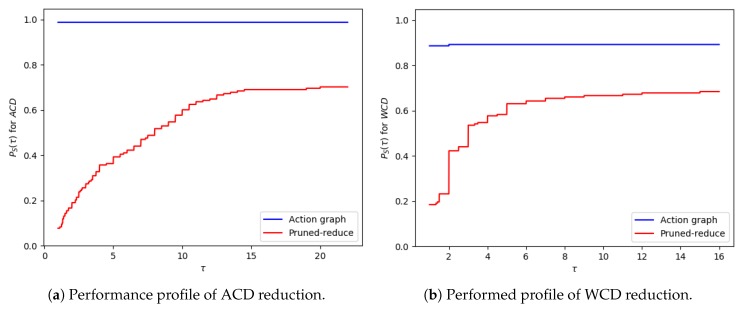
Results for comparing our Action Graph approach to pruned-reduce on all action removal experiments.

Experiments were ran on both datasets: with varying amounts of goals and with an increasing grid size. The corresponding results, showing the average time and number of actions removed, are provided in Figure 18 and Figure 19; and the WCD reduction and ACD reduction comparisons are shown in Figure 20 (separate results for the datasets are provided in Appendix E.2).

For the majority of problems, within the varying number of goals dataset, our Action Graph approach took less than 1 second to perform GRD. Whereas pruned-reduce hit the timeout for the majority of problems (Figure 18a). The execution time (i.e., minimum and maximum) varies between different problems as for some problems removing a small number of actions reduces WCD to 0, whereas for others WCD can only be partially reduced; further, the optimal plans within the different problems differ in length (longer plans take more time to find and iterate over). The same trends are observed in the results for an increasing grid size (Figure 19).

Our approach successfully managed to reduce WCD more than pruned-reduce. Pruned-reduce attempts to remove an increasing number of actions; thus, as shown in Figure 18b, did not attempt to remove many actions before the timeout was reached. This resulted in pruned-reduce not reducing the WCD as much as our approach (Figure 20b), and there were more problems for which pruned-reduce did not manage to reduce the WCD. This was also observed in the ACD reduction (Figure 20a). The aim of pruned-reduce is to reduce WCD (not ACD); thus, it performs worse at reducing ACD than WCD.

For several problems, our approach reduced ACD but not WCD. This indicates that although the least distinctive goal could not be made more distinctive, some goals could be. Both metrics provide an important insight into the distinctiveness of an environment; thus, in future GRD experiments we propose providing both.

These experiments proved that our Action Graph approach is more scalable than a current state-of-the-art approach on grid-based navigation problems. Our approach has managed to reduce the WCD of 168 grid-based navigation environments, with various numbers of goals and grid sizes, from an average of 6.29 (ACD=4.69) actions to 3.02 (ACD=1.91) actions, in an average of 4.69 s per problem.

## 10. Conclusions

As the plans to reach different goals can start with the same actions, a human’s goal often cannot be recognised until their plan nears completion. By redesigning an environment, our work enables the goal of a human to be recognised after fewer observations. This is achieved through transforming a PDDL defined Goal Recognition Design (GRD) problem into an Action Graph, by means of a Breadth First Search (BFS) from each of the goal states to the initial world state. The non-distinctive plan prefixes are then extracted to calculate how distinctive the goals are, i.e., the ${\mathrm{WCD}}_{\mathrm{dep}}$ and ${\mathrm{ACD}}_{\mathrm{dep}}$. Subsequently, these prefixes are processed to determine which actions can be replaced or removed. Our Shrink–Reduce method replaces actions by first shrinking the plans, then reducing the non-distinctive prefixes. Shrink–Reduce is less computational expensive than an exhaustive approach; however, when ran on kitchen domain Shrink–Reduce only reduces the ${\mathrm{ACD}}_{\mathrm{dep}}$ by 1, whereas exhaustive reduces ${\mathrm{ACD}}_{\mathrm{dep}}$ by 1.67. Our action removal method is shown to increase the distinctiveness of various grid-based navigation problems, with a width/height ranging from 4 to 16 and between 2 to 14 randomly selected goals, by an average of 3.27 actions in an average time of 4.69 s, whereas a state-of-the-art approach, namely pruned-reduce [10], often breaches a 10 min time limit.

Action Graphs are acyclic and no actions are repeated, therefore domains in which an action has different dependencies under varying circumstances are not currently solvable by our approach. For instance, if to reach one goal a human must retrieve item1 before taking item2, but to reach another goal the human must take item3 before item2, the action to retrieve item2 has different dependencies. For many domains, e.g., the kitchen domain, this strict ordering is not required, as humans are unlikely to adhere to a fixed ordering of actions, but for other domains this may be required. In future work, we will consider enabling actions to be repeated within an Action Graph. For domains such as the barman domain [43], in which different cocktails are created, a combination of our unconstrained method (as ingredients can be added in any order) and constrained method (as grasping and leaving a shot requires an optimal plan) will be required.

In future work we intend to apply our Action Graph approach to other, closely related, research domains. For instance, GRD with stochastic actions [14,44], plan recognition design [21], goal recognition [8] and task planning (e.g., plan legibility [45,46]). Future experiments will hopefully demonstrate how Action Graphs can help all agents in collaborative smart environments to become more aware of each other’s intentions.

Further to contextual-awareness in human-inhabited environments, our work is applicable to numerous application areas. In human computer interaction scenarios, detecting a network intruder’s intentions [21,26,47] or offering a user assistance [48,49] can also benefit from recognising the user’s goal sooner. In video game development [50] often the world is designed so the player’s goal can be recognised, thus enabling the non-playable characters to assist or thwart them; moreover these characters’ plans can also be modelled as an Action Graph. Action Graphs could allow robots to learn from humans. For instance, the graph could be built from observations or their structure adjusted to match the order humans perform actions. We hope this paper will inspire researchers in these domains, to incorporate our Action Graph approach into their work.

## Figures and Tables

**Table 1:** List of state change actions returned by the Shrink–Reduce and Exhaustive methods, that when applied to the initial state (*I*) result in the designed environment. The initial ${\mathrm{WCD}}_{\mathrm{dep}}=14$, ${\mathrm{ACD}}_{\mathrm{dep}}=11.00$, WCD=7.

**Shrink–Reduce:**	**Exhaustive:**
$WCD_{dep}=12$, $ACD_{dep}=10.00$, WCD=6	$WCD_{dep}=11$, $ACD_{dep}=9.33$, WCD=6
move-item(bread cupboard1 cupboard2)	move-item(bread cupboard1 cupboard2)
move-item(water-jug cupboard2 cupboard1)	move-item(water-jug cupboard2 cupboard1)
move-item(bowl cupboard2 cupboard1)	move-item(cup cupboard2 cupboard1)

**Figure A1:**
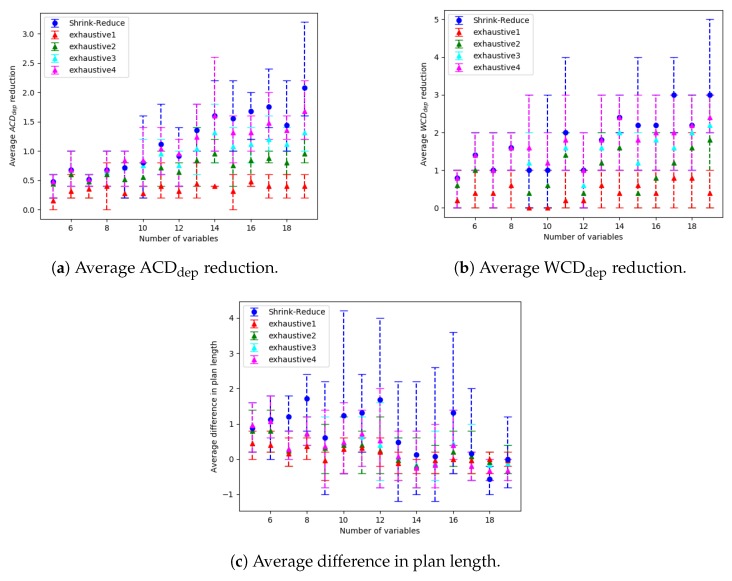
Results for an increasing number of variables.

**Figure A2:**
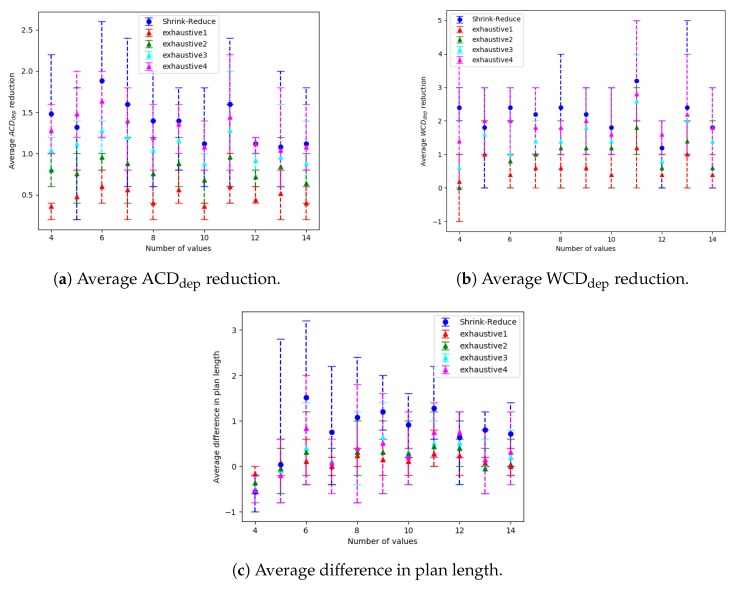
Results for an increasing number of values.

**Figure A4:**
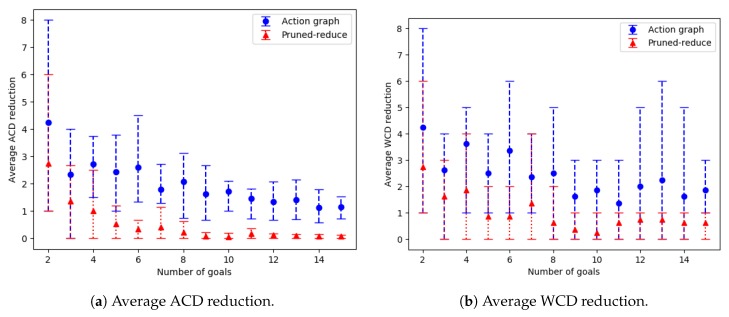
Results for an increasing number of goals. The results of our Action Graph approach are indicated by blue circles, pruned reduce [10] is shown with red triangles.

**Figure A5:**
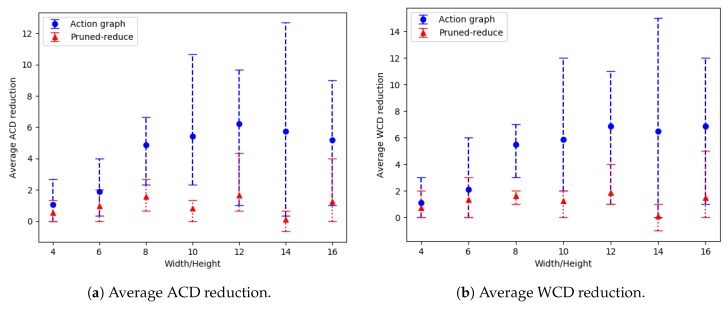
Results for an increasing grid size. The results of our Action Graph approach are indicated by blue circles, pruned-reduce [10] is shown with red triangles.

## Supplementary Materials

The following are available at https://www.mdpi.com/1424-8220/19/12/2741/s1. Video S1: Solving GRD Problems with Shrink–Reduce, Video S2: GRD for Navigation Domains.

## Abbreviations

The following abbreviations are used in this manuscript:

AI	Artifical Intelligence
IoT	Internet of Things
GR	Goal Recognition
GRD	Goal Recognition Design
WCD	Worst Case Distinctiveness
ACD	Average distinctiveness over all goals’ worst cases
BFS	Breadth First Search
PDDL	Planning Domain Definition Language

## Appendix A. Breadth First Search for Constrained Problems

Action Graphs are created by running a BFS search from each goal state to the initial state. Pseudo-code to create the Action Graph of navigation problems is shown in Algorithm A1. For such problems only the optimal plans for each goal are inserted into the graph. For other domains, in which all plan variations (including sub-optimal plans) are inserted into the graph, the limit variable is not set and goals may contain multiple atoms (e.g., line 12 becomes $a\in \{a^{\prime }|a_{eff}^{\prime }\subseteq G\}$).

**Algorithm A1** BFS for generating an Action Graph containing optimal plans
> **Inputs**: list of goals, list of actions and the initial state
> **Output**: the Action Graph
1: **function** GENERATE_ACTION_GRAPH(G, *A*, *I*) ▹ Inputs: list of goals, list of actions and the initial state
2: **for each** G∈G **do**
3: limit=−1
4: **for** $a\in \{a^{\prime }|a_{eff}^{\prime }=G\}$ **do**
5: $graph.insert\_action\_and\_deps(a,\left\{a^{\prime }|a_{pre}=a_{eff}^{\prime }\right\})$ ▹ Insert action and its dependencies
6: **if** $a_{pre}\subset I$ **then**
7: limit=1
8: **else**
9: q.push_back(a) ▹ Initialise the BFS queue, but only if *a* was not already in the graph
10: **end if**
11: **end for**
12: **while** q≠∅ **do**
13: a=q.pop()
14: **for** $d\in \{a^{\prime }|a_{pre}=a_{eff}^{\prime }\}$ **do**
15: **if** $d_{pre}\subseteq I$ **or** any $\left\{d^{\prime }|d_{pre}=d_{eff}^{\prime }\right\}$ have been inserted when processing previous goal(s) **then**
16: limit= max distance from goal
17: **else if** distance(G,d)=limit **or** all $\left\{d^{\prime }|d_{pre}=d_{eff}^{\prime }\right\}$ inserted when processing *G* **then**
18: graph.remove(d) ▹ Remove action and dependant actions whose branches will not reach *I*
19: **else**
20: $graph.insert\_action\_and\_deps(d,\left\{d^{\prime }|d_{pre}=d_{eff}^{\prime }\right\})$
21: q.push_back(d)
22: **end if**
23: **end for**
24: **if** $a_{pre}$ not met by actions within the graph **then** ▹ failed to insert an action to reach ≥1 of $a_{pre}$
25: graph.remove(a)
26: **end if**
27: **end while**
28: **end for**
29: **end function**

## Appendix B. Example Action Definition for Action Replacement

The modifications to the state of the environment, that can be made during the action replacement process, are defined as PDDL actions. In the example provided in Listing A1, an object (e.g., cup) is moved from one container (e.g., a cupboard) to another. Objects cannot be removed from the fridge or drawer (i.e., (not (= ?s fridge)), (not (= ?s drawer))), and no object can be moved into the fridge or drawer (i.e., (not (= ?g fridge)), (not (= ?g drawer))).

**Listing A1.** Example PDDL actions for action replacement.
(:action move-item-state-modification :parameters (?obj - object ?s - container ?g - container) :precondition (and (in ?obj ?s) (not (in ?obj ?g)) (not (= ?s fridge)) (not (= ?g fridge)) (not (= ?s drawer)) (not (= ?g drawer)) ) :effect ( and (in ?obj ?g) (not (in ?obj ?s)) ) )

## Appendix C. Exhaustive Algorithm for Reducing ACD

The exhaustive action replacement algorithm iteratively applies each modification to the Action Graph, then pairs of modifications, then triples, etc. The pseudo-code is shown in Algorithm A2.

**Algorithm A2** Reduce ${\mathrm{ACD}}_{\mathrm{dep}}$: Exhaustive
> **Inputs**: maximum number of modification, action graph and list of actions to modify the initial state
> **Output**: list of modifications (actions) to produce the resulting environment design
1: **function** exhaustive(N,graph,stateModifications)
2: lowestAcd=graph.calculate_acd()
3: **for** n=1;n<N;n++ **do**
4: mods=get_first_valid_combination(stateModifications,n)
5: **do**
6: graph.apply_all_modifications(mods) ▹ i.e., replaces actions affected by the modifications with their replacements
7: acd=graph.calculate_acd()
8: **if** acd<lowestAcd **then**
9: lowestAcd=acd
10: bestMods=mods
11: **end if**
12: graph.undo_all_modifications(mods)
13: **while** get_next_valid_combination(stateModifications,n,mods)
14: **end for**
15: **return** bestMods
16: **end function**

## Appendix D. Plans Generated from the Kitchen Domain

During the experiments, our action replacement algorithms were ran on a kitchen domain, to increase the distinctiveness of the plans for making breakfast, a packed-lunch and dinner. In this Appendix we provide further details on the plans producible from this domain. This domain was originally developed by Ramírez and Geffner [25]; we expanded it to include container objects, an open container action definition and a take item from container action definition. A single permutation of the longest plan to each of the goals within the kitchen domain are provided, in Table A1. There also exits plans to:

- make-dinner() with either just make-salad or make-cheese-sandwich
- make-breakfast() with make-coffee() instead of make-tea(). In addition, the option of making tea without milk and sugar or with just sugar.
- pack-lunch() with make-cheese-sandwich() instead of make-peanut=butter-sandwich()

## Appendix E. Detailed Results Graphs

In the experiments section the performance profiles for the reduction in ${\mathrm{ACD}}_{\mathrm{dep}}$ and ${\mathrm{WCD}}_{\mathrm{dep}}$ are shown. In this Appendix graphs showing the average reduction for each dataset are provided independently. These provide further detail on the performs of the different approaches. The graphs for the action replacement experiments are shown, followed by the action removal graphs. All error bars show the minimum and maximum result.

**Table A1:** Longest plans for the goals from the Kitchen domain.

(made_breakfast)	(packed_lunch)	(made_dinner)
open(cupboard1)	open(cupboard1)	open(cupboard1)
open(cupboard2)	open(cupboard2)	open(fridge)
open(drawer)	open(drawer)	open(cupboard2)
open(fridge)	open(fridge)	take(salad-tosser cupboard2)
take(kettle)	take(peanut-butter fridge)	take(bowl cupboard2)
take(cloth)	take(bread cupboard1)	take(plate cupboard2)
take(tea-bag cupboard1)	take(knife drawer)	take(dressing fridge)
take(sugar cupboard1)	take(plate cupboard2)	take(cheese fridge)
take(cereal cupboard1)	take(lunch-bag cupboard2)	take(bread cupboard1)
take(bread cupboard1)	make-peanut-butter-sandwich()	make-salad()
take(water-jug cupboard2)	pack-lunch()	make-cheese-sandwich()
take(cup cupboard2)		make-dinner
take(bowl cupboard2)		
take(knife drawer)		
take(spoon drawer)		
take(butter fridge)		
take(milk fridge)		
make-cereal()		
make-toast()		
boil-water()		
make-tea()		
use(toaster)		
make-toast()		
make-buttered-toast()		
make-breakfast()		

### Appendix E.1. Action Replacement

Our action replacement experiments compare our Shrink–Reduce and exhaustive methods. The exhaustive approach was ran for varying values of N, i.e., 1, 2, 3 and 4; which are named exhaustive1, exhaustive2, exhaustive3 and exhaustive4 in the results. A test domain was developed containing actions to open containers and take items; the state of the environment can be modified by changing which container items are in. Experiments were ran on datasets with a varying number of variables (items), values (containers) and goals. The corresponding detailed results, showing the average ${\mathrm{ACD}}_{\mathrm{dep}}$ reduction, ${\mathrm{WCD}}_{\mathrm{dep}}$ reduction and the impact (difference) in average plan length the resulting environment design has, are provided in Figure A1, Figure A2 and Figure A3. As the datasets contains an element of randomness, all points on the graphs shown the average result for 5 problems.

### Appendix E.2. Action Removal

The action removal experiments compare our approach to the pruned-reduce method by Keren et al. [10]. A grid-based navigation domain was provided as input. The average ACD and WCD reduction for an increasing number of goals and varying grid sizes (with equal width and height) are provided in Figure A4 and Figure A5. As the datasets contains an element of randomness, all points on the graphs shown the average result for 8 problems.
